# ESI-MS Study of the Interaction of Potential Oxidovanadium(IV) Drugs and Amavadin with Model Proteins

**DOI:** 10.1021/acs.inorgchem.0c00969

**Published:** 2020-06-25

**Authors:** Valeria Ugone, Daniele Sanna, Giuseppe Sciortino, Debbie C. Crans, Eugenio Garribba

**Affiliations:** † Dipartimento di Chimica e Farmacia, Università di Sassari, Via Vienna 2, I-07100 Sassari, Italy; ‡ Istituto CNR di Chimica Biomolecolare, Trav. La Crucca 3, I-07040 Sassari, Italy; § Departament de Química, Universitat Autònoma de Barcelona, 08193 Cerdanyola del Vallés, Barcelona, Spain; ∥ Department of Chemistry, Colorado State University, 1301 Center Avenue, Fort Collins, Colorado, United States

## Abstract

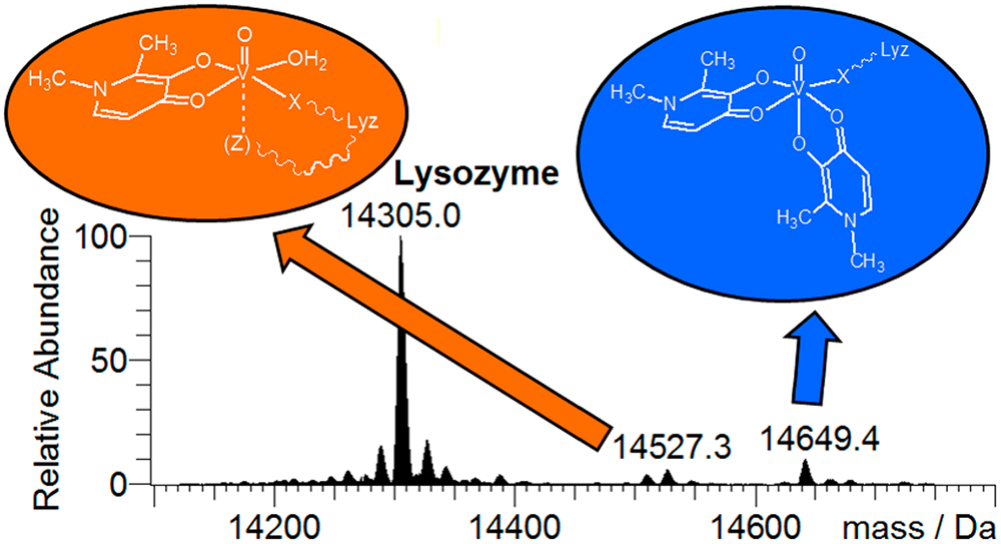

In this study, the binding to lysozyme (Lyz) of four important V^IV^ compounds with antidiabetic and/or anticancer activity, [V^IV^O(pic)_2_(H_2_O)], [V^IV^O(ma)_2_], [V^IV^O(dhp)_2_], and [V^IV^O(acac)_2_], where pic^–^, ma^–^, dhp^–^, and acac^–^ are picolinate, maltolate, 1,2-dimethyl-3-hydroxy-4(1*H*)-pyridinonate, and acetylacetonate anions, and of the vanadium-containing natural product amavadin ([V^IV^(hidpa)_2_]^2–^, with hidpa^3–^*N*-hydroxyimino-2,2′-diisopropionate) was investigated by ElectroSpray Ionization-Mass Spectrometry (ESI-MS). Moreover, the interaction of [V^IV^O(pic)_2_(H_2_O)], chosen as a representative V^IV^O^2+^ complex, was examined with two additional proteins, myoglobin (Mb) and ubiquitin (Ub), to compare the data. The examined vanadium concentration was in the range 15–150 μM, i.e., very close to that found under physiological conditions. With pic^–^, dhp^–^, and hidpa^3–^, the formation of adducts *n*[V^IV^OL_2_]–Lyz or *n*[V^IV^L_2_]–Lyz is favored, while with ma^–^ and acac^–^ the species *n*[V^IV^OL]–Lyz are detected, with *n* dependent on the experimental V^IV^/protein ratio. The behavior of the systems with [V^IV^O(pic)_2_(H_2_O)] and Mb or Ub is very similar to that of Lyz. The results suggested that under physiological conditions, the moiety *cis*-V^IV^OL_2_ (L = pic^–^, dhp^–^) is bound by only one accessible side-chain protein residue that can be Asp, Glu, or His, while V^IV^OL^+^ (L = ma^–^, acac^–^) can interact with the two equatorial and axial sites. If the V^IV^ complex is thermodynamically stable and does not have available coordination positions, such as amavadin, the protein cannot interact with it through the formation of coordination bonds and, in such cases, noncovalent interactions are predicted. The formation of the adducts is dependent on the thermodynamic stability and geometry in aqueous solution of the V^IV^O^2+^ complex and affects the transport, uptake, and mechanism of action of potential V drugs.

## Abstract 2

The potentiality of ElectroSpray Ionization-Mass Spectrometry (ESI-MS) to determine the number and composition of adducts formed by labile V complexes with potential pharmacological application and the natural product amavadin with some proteins was examined. ESI-MS allows the study of vanadium concentrations close to those found under physiological conditions. The nature of the adducts—which could be related to the transport, uptake, and mechanism of action of potential V drugs—depends on the structure and thermodynamic stability at concentrations around micromolar.

## Introduction

Vanadium compounds (VCs) show a wide variety of pharmacological actions, among which are antiviral, antiparasitic, and antituberculosis effects, even though the most studied and promising application in medicine could be the treatment of diabetes and some types of cancer.^1^ The study of the biospeciation of potential vanadium drugs is of fundamental importance to the prediction of (i) the form in which they are transported to the target organs, (ii) which is the active species in the organism, and (iii) the mechanism of action and binding to the biological receptors. In this context, the interaction of antidiabetic and anticarcinogenic VCs with the main components of the blood, including proteins, has been widely investigated.^2,3^ In particular, proteins play a central role in the biospeciation and biotransformation of a VC in the organism, for both their high affinity toward V and high concentration in biological fluids.

In addition to X-ray diffraction, alternative techniques to obtain information on the metal–protein adducts are desirable; among them, NMR, EPR, ESEEM, ENDOR, UV–vis, and CD spectroscopy have been applied to systems containing V^III^, V^IV^, or V^V^.^4^ More recently, other methods, such as voltammetry and polarography, HPLC-ICP-MS, size-exclusion chromatography, gel-electrophoresis, and MALDI-TOF were used in combination with the other techniques.^3o,5^ All these tools present some limitations: for example, NMR can be used mainly with V^V^, and EPR—the spectroscopy most frequently used for paramagnetic vanadium(IV) compounds^6^—provides information on the type of residues involved in the metal binding but less on the stoichiometry of the formed adducts. Moreover, the relatively high vanadium concentration necessary to ensure a good signal-to-noise ratio in NMR and EPR measurements exceeds by several orders of magnitude that found in healthy humans (0.2–15 nM^1e,7^) and in the patients treated with V drugs (1–10 μM for inorganic salts^1b,3a,8^ and 40–60 μM when complexes such as bis(maltolato)oxidovanadium(IV) (BMOV) or bis(ethylmaltolato)oxidovanadium(IV) (BEOV) are administered to rats^3m,9^). Therefore, alternative techniques that are more sensitive at the physiological concentrations are needed.

Over the past decade, the potentiality of mass spectrometry (MS) to study the behavior of metallodrugs in biological samples and characterize at the molecular level their interaction with biomolecules and potential targets, such as proteins, has been discussed.^10,11^ Among the instrumental techniques based on MS, ESI (ElectroSpray Ionization) and MALDI (Matrix-Assisted Laser Desorption/Ionization) are very powerful methods to ascertain metallodrug–protein binding. While MALDI generates mainly singly charged pseudomolecular ions, ESI gives singly or multiply charged metal–protein adducts (upon deprotonation or association with protons and alkali ions).^10c,11^ For example, ESI-MS has been used to explore the interaction of cisplatin and its derivatives to low molecular mass bioligands, such as DNA nucleic bases, amino acids, oligonucleotides, and peptides, and high molecular mass biomolecules, such as proteins;^10a,10c,12^ moreover, the study of the reactivity of organometallic complexes of Ru^II^ and Au^III^ with potential antitumoral applications is another example of its potentiality.^10b,13,14^ An important advantage over other instrumental techniques is that the metal concentrations for ESI-MS experiments are in the range 1–100 μM, therefore very close to those found under physiological conditions. Among others, lysozyme, ubiquitin, myoglobin, superoxido dismutases, and insulin have been extensively studied by ESI-MS as model proteins;^11^ they have structural features which make them ideal compounds for MS experiments: a small or moderate size (with molecular masses ranging from 5500 to 33000 Da) and high stability in solution under physiological-like conditions, commercial availability with high purity, water-solubility, and easy ionizability. Further ESI-MS studies have been carried out with potential molecular targets, such as metallothioneins, glutathione-*S*-transferases, cytochrome *c*, and calmodulin.^11^ Model studies with the above-mentioned proteins can be very useful, since the proteins involved in the transport and mechanism of action of a metallodrug (albumin and transferrin, for example) are not available in a purified form and have often high molecular masses.^10c^

Until now, the ESI technique was applied to inert metal complexes of the second and third transition series in which proteins bind with a coordination bond.^11^ However, it has been pointed out that such a technique can also be used to study metal species which form labile coordinative bonds with biomolecules (such as vanadium, copper, and other metals of the first transition row) or to systems where noncovalent binding occurs (i.e., coordinatively saturated, such as Pt^IV^ complexes).^11,15^ Despite the high number of articles published on Pt, Ru, and Au complexes with antitumoral activity,^11^ very few papers have been devoted to potential V drugs: beyond the “classical” applications of MS in the determination of the molecular mass of synthetic V complexes, the number of other studies is rather limited. One example is the ICP-MS investigation of the biospeciation of some antidiabetic V^IV^O^2+^ compounds in the blood serum,^16^ in which the binding of VCs to serum proteins, in particular transferrin, was suggested.

**Scheme 1:**
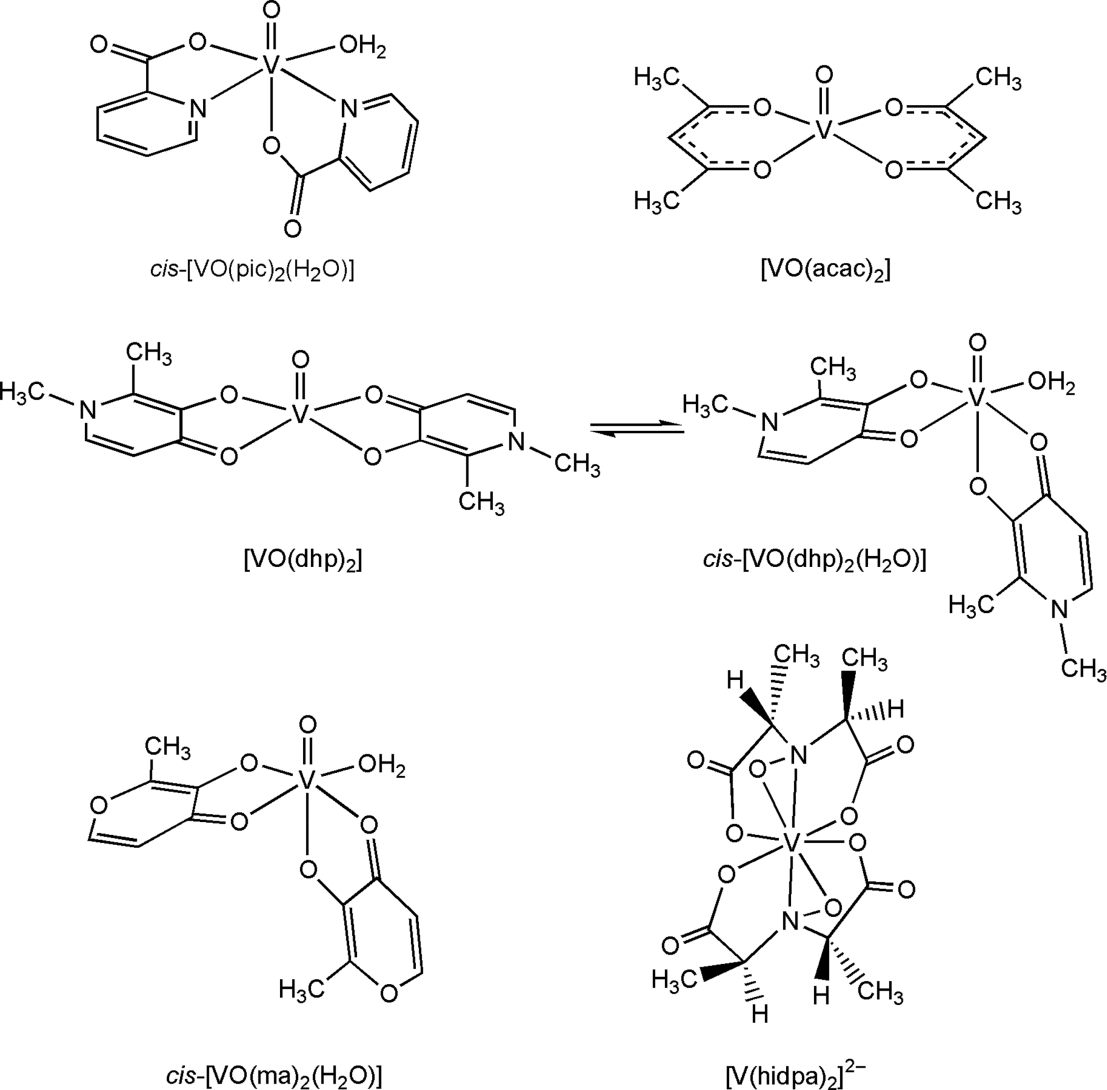
Structure in Aqueous Solution of the VCs Studied in This Work: *cis*-[V^IV^O(pic)_2_(H_2_O)], [V^IV^O(acac)_2_], [V^IV^O(dhp)_2_] ⇄ *cis*-[V^IV^O(dhp)_2_(H_2_O)], *cis*-[V^IV^O(ma)_2_(H_2_O)], and [V^IV^(hidpa)_2_]^2–^ (amavadin) The charges of the ligands and V=O ion (2+) are omitted for clarity.

In this study, the interaction of V^IV^ compounds with pharmacological and biological relevance with some model proteins has been investigated by ESI-MS spectrometry. In particular, the binding to lysozyme (Lyz) with four important VCs with potential antidiabetic or anticancer application,^1e,3m^ [V^IV^O(pic)_2_(H_2_O)], [V^IV^O(ma)_2_], [V^IV^O(dhp)_2_], and [V^IV^O(acac)_2_], where pic^–^, ma^–^, dhp^–^, and acac^–^ are the abbreviations for picolinate, maltolate, 1,2-dimethyl-3-hydroxy-4(1*H*)-pyridinonate, and acetylacetonate, and with [V(hidpa)_2_]^2–^ (amavadin, with hipda^3–^*N*-hydroxyimino-2,2′-diisopropionate anion^4,17^) was investigated (Scheme 1). [V^IV^O(pic)_2_(H_2_O)], [V^IV^O(ma)_2_], and [V^IV^O(dhp)_2_] exist in aqueous solution, at least in part, as *cis*-octahedral species (with *cis* referring to the position of the water molecule with respect to the V=O bond, Scheme 1) with an accessible site in the equatorial position where an amino acid side-chain donor can replace the water ligand. In contrast, [V^IV^O(acac)_2_] is square pyramidal and amavadin is an unusual nonoxido V^IV^ species with no available coordination sites. Subsequently, the interaction of [V^IV^O(pic)_2_(H_2_O)], chosen as a representative V^IV^O^2+^ species, was examined with myoglobin (Mb) and ubiquitin (Ub), to compare the behavior of different proteins. The main goal of this work is to ascertain if the ESI-MS could be considered as a valid technique to infer information on the VCs–protein interactions, trying to highlight the pros and cons, and to understand its possible applications in the design and development of new potential V drugs.

## Experimental Section

### Chemicals

The chemicals oxidovanadium(IV) sulfate trihydrate (V^IV^OSO_4_·3H_2_O), pyridine-2-carboxylic acid (picolinic acid, Hpic), 3-hydroxy-2-methyl-4*H*-pyran-4-one (maltol, Hma), 1,2-dimethyl-3-hydroxy-4(1*H*)-pyridinone (deferiprone, Hdhp), *N*-hydroxyimino-2,2′-diisopropionic acid (H_3_hidpa), lysozyme from chicken egg white (abbreviated with Lyz; code 62970), myoglobin from equine heart (abbreviated with Mb; M1882), and ubiquitin from bovine erythrocytes (abbreviated with Ub; U6253), are all Sigma-Aldrich products and were used without further purification.

The complexes [V^IV^O(dhp)_2_], [V^IV^O(pic)_2_(H_2_O)], and [V^IV^O(ma)_2_] were synthesized following the procedure established in the literature.^18^ [V^IV^O(acac)_2_] was purchased from Sigma-Aldrich. The calcium salt of amavadin, [Ca(H_2_O)_5_][V^IV^(hidpa)_2_]·H_2_O, was prepared according to the reported synthesis.^17^

### Preparation of the Solutions and ESI-MS Measurements

The solutions were prepared dissolving in ultrapure water (LC-MS grade, Sigma-Aldrich) a weighted amount of the VC to obtain a metal ion concentration of (1.0–2.0) × 10^–3^ M. They were subsequently diluted in ultrapure water and mixed with aliquots of a stock protein solution (500 μM) in order to have a metal-to-protein molar ratio of 3:1, 5:1, or 10:1 with a final protein concentration of 5 or 50 μM. In all the solutions, pH was in the range 5.0–6.0. Argon was bubbled through the solutions to ensure the absence of oxygen and avoid the oxidation of the V^IV^O^2+^ ion. ESI-MS spectra were recorded immediately after the solution preparation.

Mass spectra in positive- and negative-ion mode were obtained on a Q Exactive Plus Hybrid Quadrupole-Orbitrap (Thermo Fisher Scientific) mass spectrometer. The solutions were infused at a flow rate of 5.00 μL/min into the ESI chamber. The spectra were recorded in the *m*/*z* range 50–750 for the binary and 300–4500 for the ternary systems at a resolution of 140 000 and accumulated for at least 5 min to increase the signal-to-noise ratio. Generally the experiments were run more than once; when measurements were repeated, indistinguishable results were obtained. Only one set of the reproducible data is reported here.

The experimental settings for the measurements of positive-ion mode spectra were: spray voltage, 2300 V; capillary temperature, 250 °C; sheath gas, 10 (arbitrary units); auxiliary gas, 3 (arbitrary units); sweep gas, 0 (arbitrary units); probe heater temperature, 50 °C. The settings used for negative-ion mode spectra were: spray voltage, −1900 V; capillary temperature, 250 °C; sheath gas, 20 (arbitrary units); auxiliary gas, 5 (arbitrary units); sweep gas, 0 (arbitrary units); probe heater temperature, 14 °C. ESI-MS spectra were analyzed with Thermo Xcalibur 3.0.63 software (Thermo Fisher Scientific), and the average deconvoluted monoisotopic masses were obtained through the Xtract tool integrated in the software.

## Results and Discussion

### Interaction of [V^IV^O(pic)_2_(H_2_O)] with Lysozyme

Lysozyme represents one of the most frequently used models to study the metallodrug–protein interactions by mass spectrometry techniques. Lyz is a relatively small protein (with mass of ca. 14300 Da) formed of 129 amino acids with an enzymatic activity and a significant relevance in a number of host defense processes.^19^ The specific structure of this enzyme relies on its stable three-dimensional conformation associated with the presence of four disulfide bonds between Cys residues alongside the peptide chain. It has only one histidine in its structure (His15), which could represent a general binding site for some transition metal complexes.^11^

**Figure 1:**
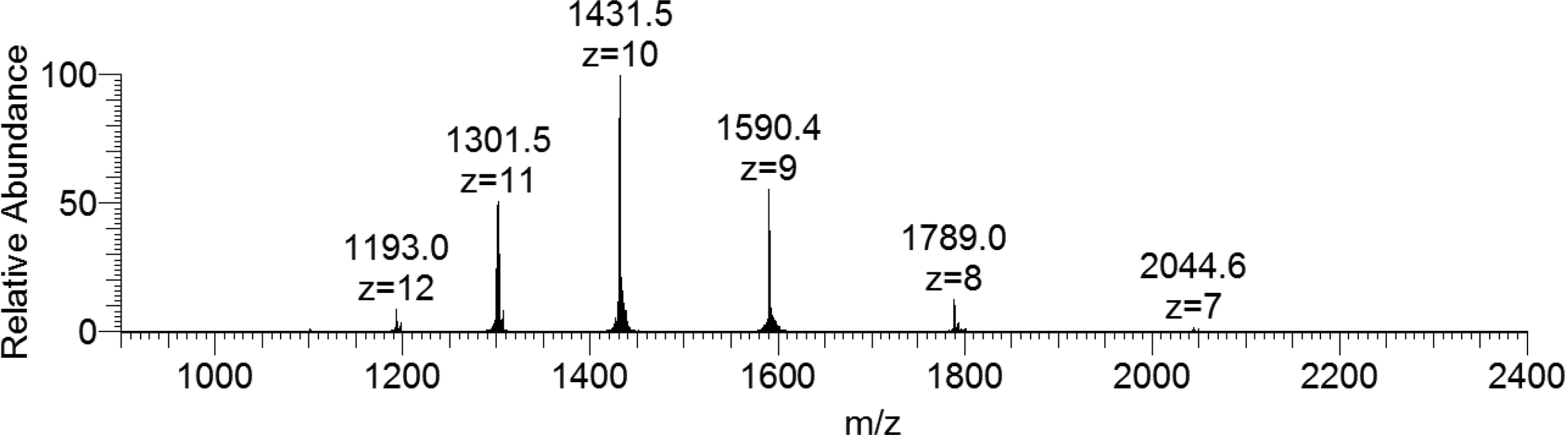
ESI-MS spectrum of lysozyme (concentration 5 μM).

**Figure 2:**
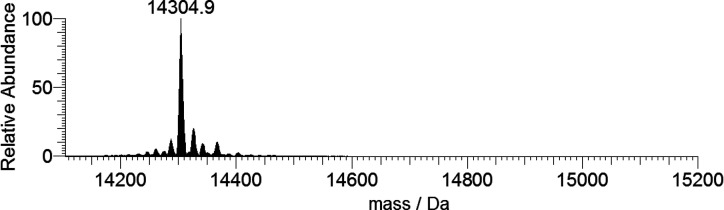
Deconvoluted ESI-MS spectrum of lysozyme (concentration 5 μM).

The positive-ion mode ESI-MS spectrum of lysozyme shows a series of well-resolved peaks with a charge distribution from *z* = 7 to *z* = 12, which fall in the *m*/*z* range of 1100–2100 (Figure 1).^20^ To determine the exact mass of the protein, the spectrum was deconvoluted with the Xtract software, which allows an estimation of the mass of macromolecules from the mass-to-charge spectra consisting of several peaks for multiply charged ions.^21^ The result is reported in Figure 2, where a central intense peak at 14305 Da can be noticed with a series of other signals due to the adducts containing Na^+^ ions (23 Da) and/or H_2_O molecules (18 Da). Each peak is also split in several signals related to the isotopic distribution of the revealed species.

In order to interpret the spectra in the system containing [V^IV^O(pic)_2_(H_2_O)] and lysozyme, it must be considered that V exists in aqueous solution as a mixture of [V^IV^O(pic)_2_(H_2_O)] and [V^IV^O(pic)_2_(OH)]^−^, as demonstrated by the combined characterization by EPR and pH-potentiometric experiments.^22^ The positive-ion mode ESI-MS spectrum recorded in this study, after dissolution of the solid complex [V^IV^O(pic)_2_(H_2_O)] in ultrapure water at spontaneous pH (5.15), is reported in Figure S1 of the Supporting Information. The spectrum shows the presence of two intense peaks at *m*/*z* = 124.04 and *m*/*z* = 146.02 attributed to the protonated ligand, [Hpic+H]^+^, and its sodium adduct, [Hpic+Na]^+^. It is also possible to observe the signals of the H^+^ and Na^+^ adducts of [V^IV^O(pic)_2_], at *m*/*z* 311.99 and 333.98, respectively, confirming the presence in solution, under these experimental conditions, of the 1:2 V^IV^O–picolinato species; the equatorial H_2_O is not revealed, in agreement with the literature data suggesting that a weak monodentate ligand can be removed from the metal coordination sphere during the ionization process.^23,24^ The comparison between the experimental and calculated isotopic pattern for the peak of the [V^IV^O(pic)_2_+H]^+^ ion is shown as an example in Figure S2; in particular, it must be observed the coincidence of the two expected peaks due to the natural abundance of the ^13^C isotope (separated by *m*/*z* ∼ 1.00 for this adduct with charge +1) at the third decimal figure. In the negative-ion mode ESI spectrum, the signal of the complex in which the water molecule is deprotonated, [V^IV^O(pic)_2_(OH)]^−^, was observed together with the V^V^ species [V^V^O_2_(pic)_2_]^−^, respectively at *m*/*z* 327.99 and 326.98 (Figure S3). The detection of [V^V^O_2_(pic)_2_]^−^ is due to the partial oxidation of [V^IV^O(pic)_2_(OH)]^−^ in solution or in source.^25^ This oxidation process is probably favored by the similarity of the structure of *cis*-V^IV^O(OH)^−^ and *cis*-V^V^O_2_^+^ (Scheme S1). The species identified by ESI-MS spectrometry in the system with picolinate are listed in Table S1.

**Figure 3:**
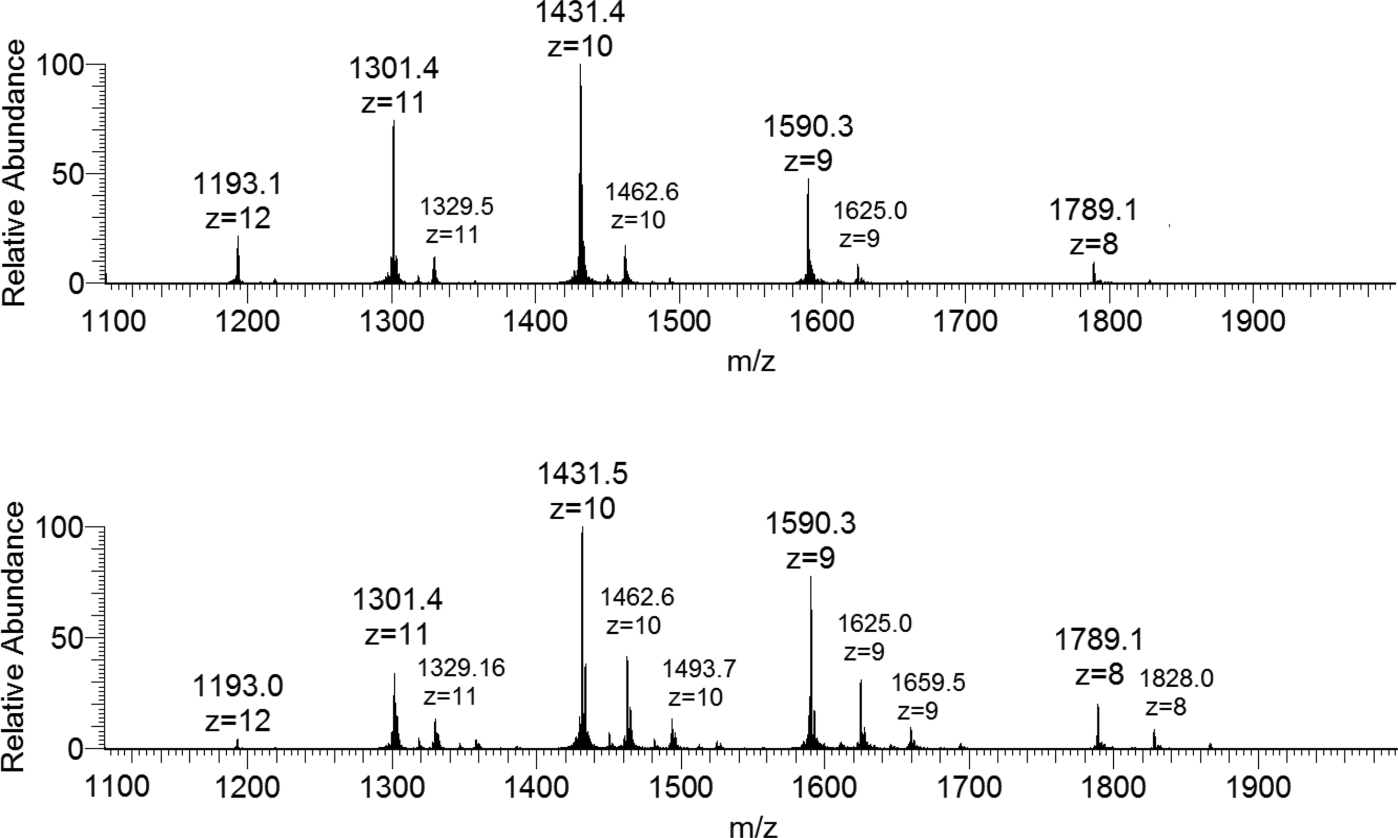
ESI-MS spectra recorded on the system containing [V^IV^O(pic)_2_(H_2_O)] and lysozyme (5 μM): molar ratios 3:1 (top) and 5:1 (bottom).

**Figure 4:**
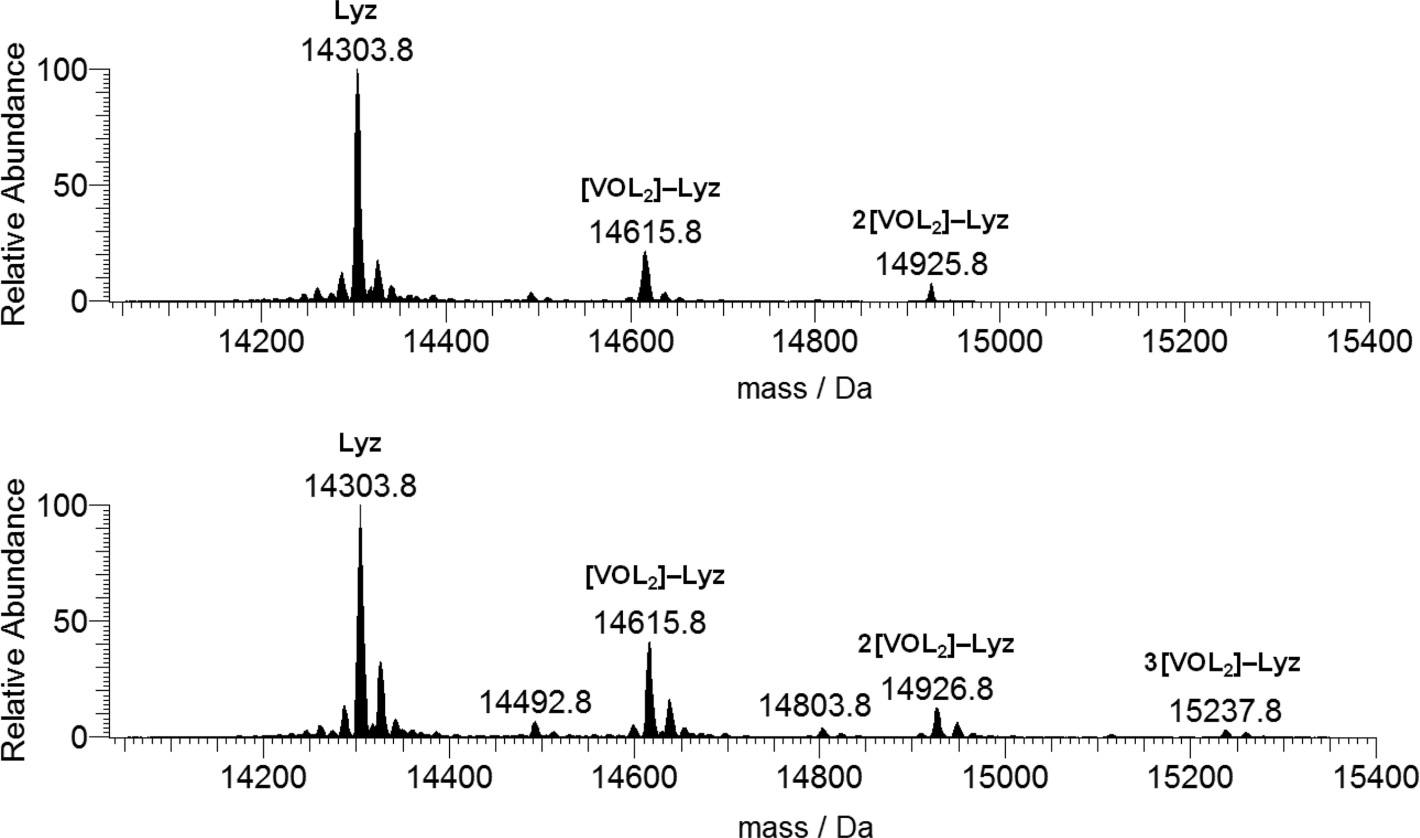
Deconvoluted ESI-MS spectra recorded on the system containing [V^IV^O(pic)_2_(H_2_O)] and lysozyme (5 μM): molar ratios 3:1 (top) and 5:1 (bottom). L indicates the picolinato ligand.

The ESI-MS spectra on the system containing [V^IV^O(pic)_2_(H_2_O)] and Lyz were recorded using two concentrations of the protein (5 and 50 μM) and metal-to-protein ratios (3:1 and 5:1). The results indicate that these two variables significantly influence the outcome of the experiments. In Figure 3, the raw spectra obtained with a protein concentration of 5 μM are represented. It can be noticed that, for each peak of the protein, other signals are present with higher *m*/*z* values, which correspond to the adducts *n*[V^IV^O(pic)_2_]–Lyz. Since the arrangement of the two ligands is equatorial–equatorial and equatorial–axial, only one equatorial coordination site is available for the protein. The comparison of the raw spectra recorded in the system [V^IV^O(pic)_2_(H_2_O)]/Lyz with those obtained in the system with lysozyme only (cfr. Figures 3 and 1) suggests that the V^IV^O(pic)_2_ binding does not alter the distribution of the protein charge states; this indicates that the conformation of lysozyme remains unchanged after the interaction with the metal moiety.^26^ To determine the mass of these adducts, the ESI-MS spectra were deconvoluted, and the results are reported in Figure 4. The spectra are similar, and in addition to the peak of the free protein, the peaks at 14 616 and 14 926 Da (for the 3:1 ratio) and also at 15 238 Da (for the 5:1 ratio) are revealed, which are attributed to the adducts [V^IV^O(pic)_2_]–Lyz, 2[V^IV^O(pic)_2_]–Lyz, and 3[V^IV^O(pic)_2_]–Lyz, in which one, two, or three *cis*-V^IV^O(pic)_2_ moieties (with mass of 311 Da) are bound to the protein.

**Figure 5:**
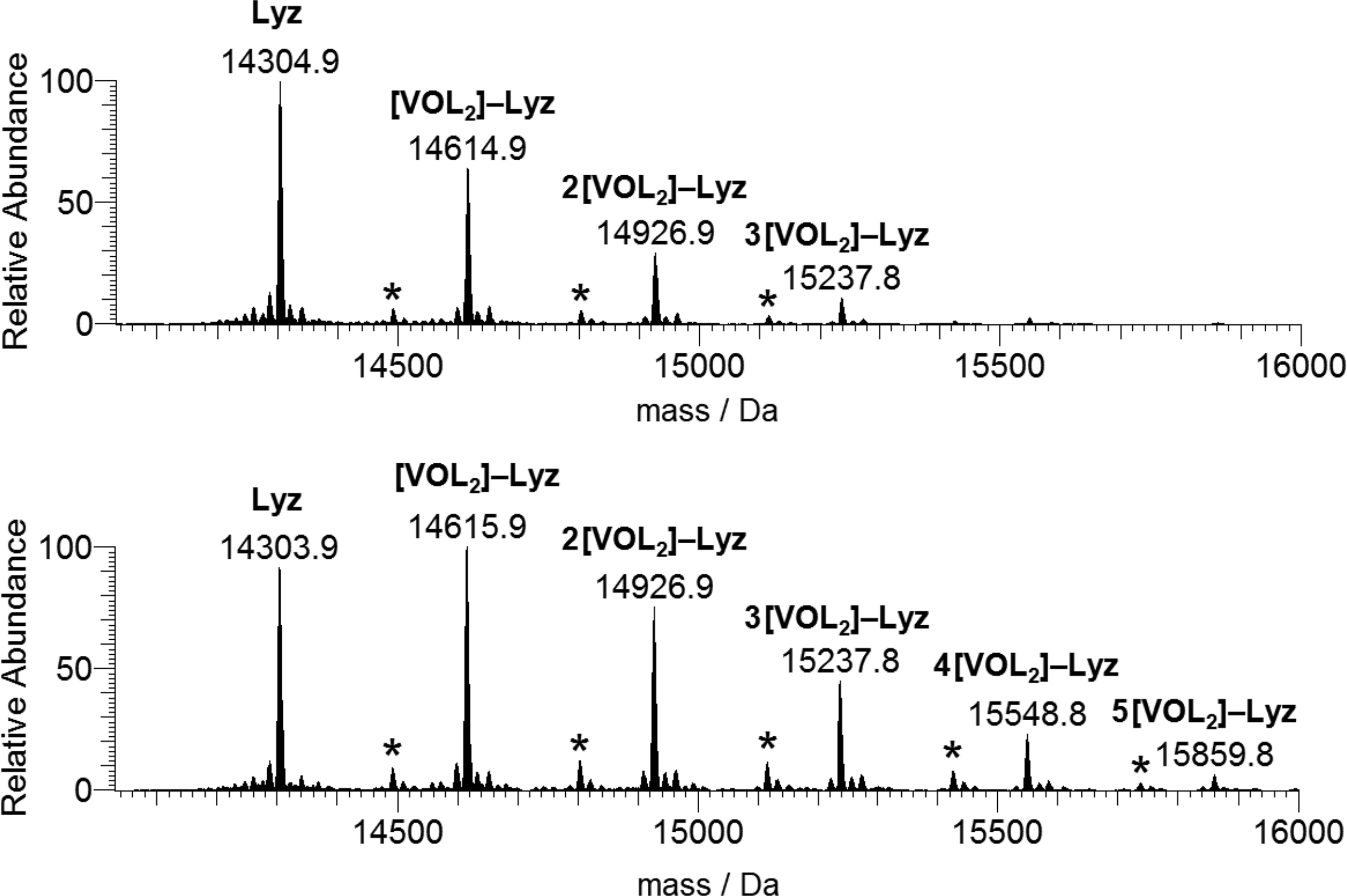
Deconvoluted ESI-MS spectra recorded on the system containing [V^IV^O(pic)_2_(H_2_O)] and lysozyme (50 μM): molar ratios 3:1 (top) and 5:1 (bottom). L indicates the picolinato ligand. With the asterisks, the peaks of the adducts {[V^IV^OL]^+^ + *n*[V^IV^OL_2_]}–Lyz with *n* = 0–2 (ratio 3:1) and *n* = 0–4 (ratio 5:1) are denoted.

If the spectra are recorded with a protein concentration of 50 μM (Figure 5), the outcome of the experiments significantly changes, and the signals of the species *n*[V^IV^O(pic)_2_]–Lyz, with *n* = 1–3 when the molar ratio is 3:1 and *n* = 1–5 when it is 5:1, are detected (Table 1). Therefore, an increase of the concentration seems to favor the interaction between the *cis*-V^IV^O(pic)_2_ moiety and lysozyme. Moreover, for each peak *n*[V^IV^O(pic)_2_]–Lyz, the signal of the adducts {[V^IV^O(pic)] + *n*[V^IV^O(pic)_2_]}–Lyz with *n* = 0–4 at the highest V/protein ratio can be observed, where both V^IV^O(pic)^+^ (with mass of 189 Da) and a certain number of *cis*-V^IV^O(pic)_2_ fragments bind to lysozyme. It must be noticed that the intensity of the signals due to the adducts with 1:1 species V^IV^O(pic)^+^ is lower than that of the adducts formed by 1:2 fragment *cis*-V^IV^O(pic)_2_.

**Table 1:** Main Adducts Formed by Pharmacologically Active VOL_2_ Complexes with Lysozyme (Lyz) Revealed by ESI-MS

compound	mass (Da)	adducts
[V^IV^O(pic)_2_(H_2_O)]	14491.8	[V^IV^O(pic)]–Lyz
	14615.8	[V^IV^O(pic)_2_]–Lyz
	14802.8	{[V^IV^O(pic)] + [V^IV^O(pic)_2_]}–Lyz
	14926.8	2[V^IV^O(pic)_2_]–Lyz
	15114.8	{[V^IV^O(pic)] + 2[V^IV^O(pic)_2_]}–Lyz
	15237.8	3[V^IV^O(pic)_2_]–Lyz
	15425.8	{[V^IV^O(pic)] + 3[V^IV^O(pic)_2_]}–Lyz
	15548.8	4[V^IV^O(pic)_2_]–Lyz
	15736.8	{[V^IV^O(pic)] + 4[V^IV^O(pic)_2_]}–Lyz
	15859.8	5[V^IV^O(pic)_2_]–Lyz
[V^IV^O(ma)_2_]	14494.8	[V^IV^O(ma)]–Lyz
	14685.8	2[V^IV^O(ma)]–Lyz
	14812.8	{[V^IV^O(ma)] + [V^IV^O(ma)_2_]}–Lyz
[V^IV^O(dhp)_2_]	14527.3	[V^IV^O(dhp)(H_2_O)]–Lyz
	14649.4	[V^IV^O(dhp)_2_]–Lyz
	14870.5	{[V^IV^O(dhp)(H_2_O)] + [V^IV^O(dhp)_2_]}–Lyz
	14992.6	2[V^IV^O(dhp)_2_]–Lyz
[V^IV^O(acac)_2_]	14470.0	[V^IV^O(acac)]–Lyz
	14571.2	[V^IV^O(acac)_2_]–Lyz
	14636.0	2[V^IV^O(acac)]–Lyz
	14736.6	{[V^IV^O(acac)] + [V^IV^O(acac)_2_]}–Lyz
	14801.0	3[V^IV^O(acac)]–Lyz
	14837.1	2[V^IV^O(acac)_2_]–Lyz
	14965.1	4[V^IV^O(acac)]–Lyz
[V(hidpa)_2_]^2–^	14706.8	[V^IV^(hidpa)_2_]–Lyz

### Interaction of [V^IV^O(ma)_2_] with Lysozyme

In the solid state, maltolate forms [V^IV^O(ma)_2_] with a square pyramidal geometry,^27^ but when the solid complex is dissolved in a coordinating solvent such as water, it undergoes isomerization to the *cis*-octahedral species [V^IV^O(ma)_2_(H_2_O)] (Scheme 1).^28^

After dissolving [V^IV^O(ma)_2_] in ultrapure water, in the positive-ion mode ESI-MS spectrum, the species [Hma+H]^+^ (*m*/*z* = 127.04) and [V^IV^O(ma)_2_+H]^+^ (*m*/*z* = 317.99, the simulation of the isotopic pattern being shown in Figure S4) were identified. In the negative-ion mode the signal of the deprotonated ligand [ma]^−^ (*m*/*z* = 125.02) and the V^V^ complex [V^V^O_2_(ma)_2_]^−^ (*m*/*z* = 332.98, simulation of the isotopic pattern in Figure S5) were detected. As previously pointed out for the system with Hpic, the detection of these species can be related to the presence in solution of [V^IV^O(ma)_2_(H_2_O)] and [V^IV^O(ma)_2_(OH)]^−^, since a solvent molecule can be lost during the ionization process and a partial oxidation of the V^IV^O^2+^ moiety can occur.^23−25^ The list of the species identified is reported in Table S2.

**Figure 6:**
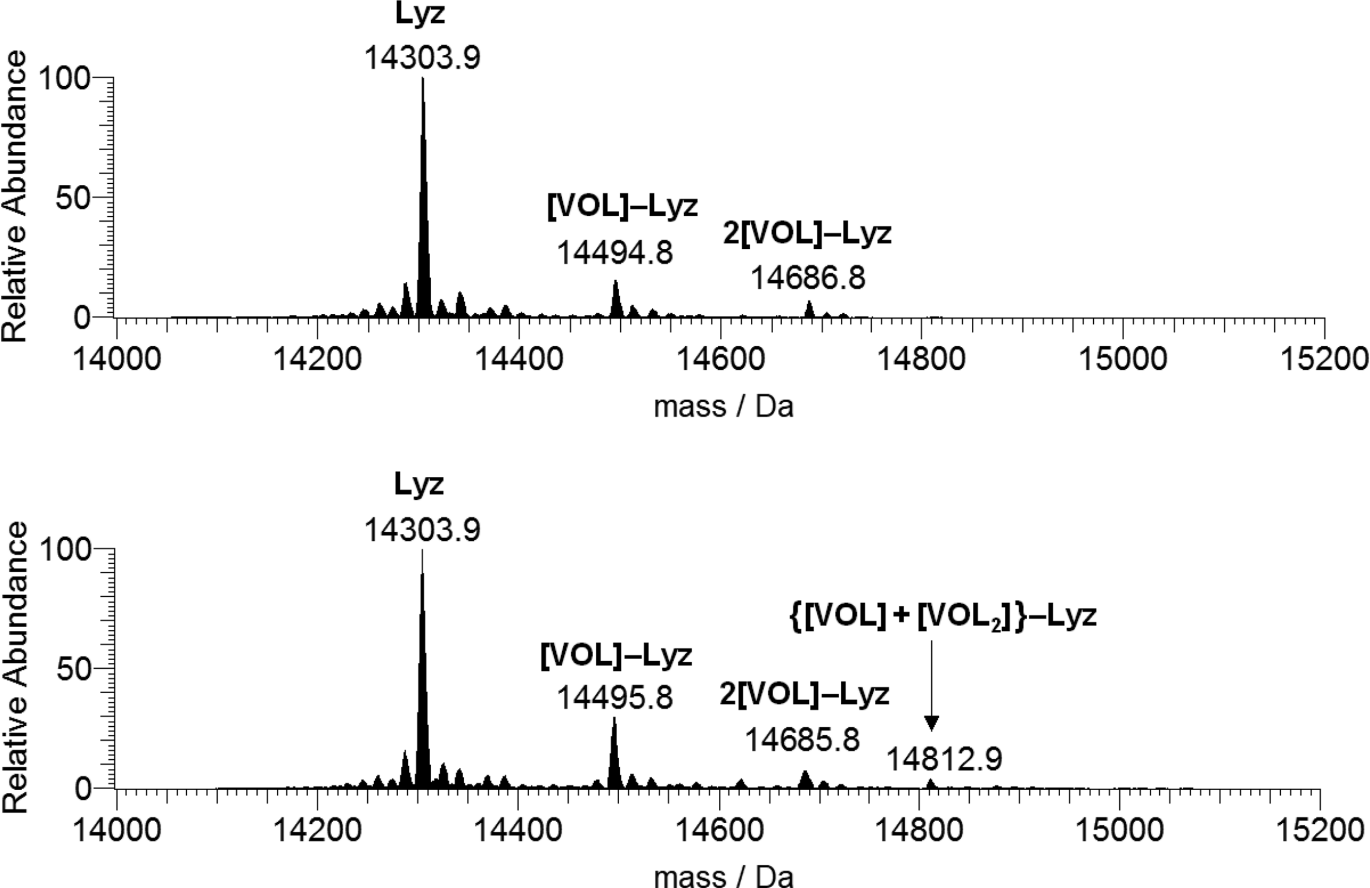
Deconvoluted ESI-MS spectra recorded on the system containing [V^IV^O(ma)_2_] and lysozyme (50 μM): molar ratios 3:1 (top) and 5:1 (bottom). L indicates the maltolato ligand.

In the ESI-MS spectra of the system [V^IV^O(ma)_2_]/Lyz recorded with a protein concentration of 5 μM and molar ratios of 3:1 and 5:1, only the signal of [V^IV^O(ma)]–Lyz (mass of V^IV^O(ma)^+^ being 191 Da) was detected (spectra not shown). However, when the lysozyme concentration is 10 times higher (i.e., 50 μM), the number of species increases, similarly to what was detected in the system with picolinate. In particular, with molar ratios 3:1 and 5:1 [V^IV^O(ma)]–Lyz (14495 Da) and 2[V^IV^O(ma)]–Lyz (14686 Da) were observed (Figure 6); moreover, a weak peak attributed to {[V^IV^O(ma)] + [V^IV^O(ma)_2_]}–Lyz (14813 Da) was revealed. It must be observed that the moieties [V^IV^O(ma)_2_] and [V^IV^O(ma)]^+^ have one and three sites (two adjacent in the equatorial plane plus the axial one) suitable for the protein binding.

Two major differences in comparison with the system with picolinate must be highlighted: (i) it is the moiety V^IV^OL^+^ instead of *cis*-V^IV^OL_2_ which preferentially binds to the protein and (ii) a smaller number of V^IV^O(ma)^+^ fragments compared to *cis*-V^IV^O(pic)_2_ interact with lysozyme with varying both the ratio and concentration.

### Interaction of [V^IV^O(dhp)_2_] with Lysozyme

In the binary system V^IV^O^2+^/Hdhp at a metal-to-ligand molar ratio of 1:2, V^IV^O^2+^ forms species with composition 1:1 and 1:2. The 1:2 complex exists, from pH 5 to 8, as a mixture of [V^IV^O(dhp)_2_(H_2_O)], *cis*-octahedral, and [V^IV^O(dhp)_2_], square pyramidal, in equilibrium between each other (Scheme 1).^18a,29^ In the ESI-MS spectrum obtained after dissolving [V^IV^O(dhp)_2_] in water, the signals at *m*/*z* 344.06 of [V^IV^O(dhp)_2_+H]^+^ and at *m*/*z* 366.04 of [V^IV^O(dhp)_2_+Na]^+^ (Figure S6) are detected, confirming the existence of the 1:2 complex in solution. The species revealed are listed in Table S3.

**Figure 7:**
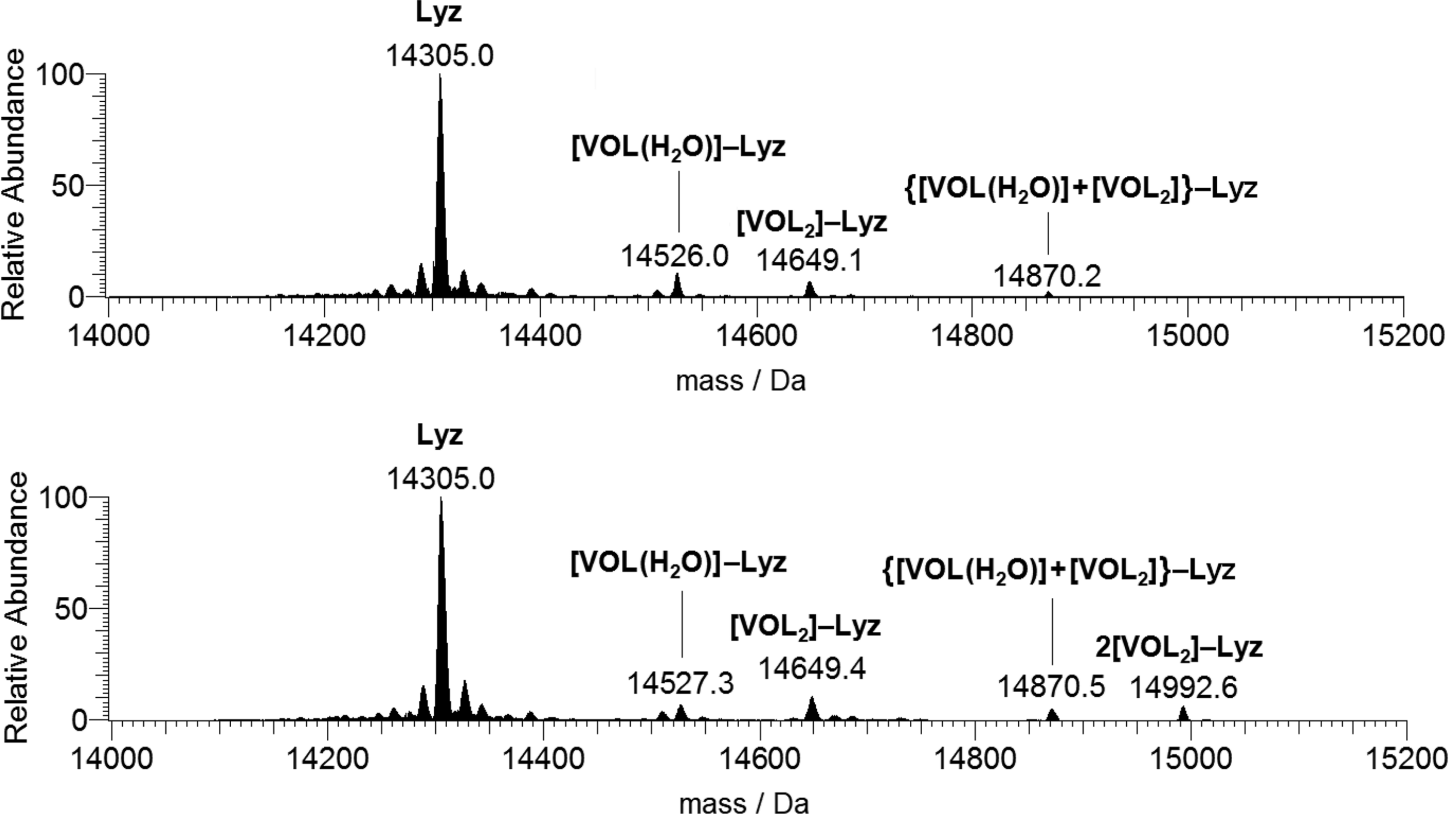
Deconvoluted ESI-MS spectra recorded on the system containing [V^IV^O(dhp)_2_] and lysozyme (5 μM): molar ratios 3:1 (top) and 5:1 (bottom). L indicates the 1,2-dimethyl-3-hydroxy-4(1*H*)-pyridinonato ligand.

In the ESI-MS spectra recorded on the system [V^IV^O(dhp)_2_]/Lyz 3:1, the species observed were [V^IV^O(dhp)(H_2_O)]–Lyz (14526 Da) and [V^IV^O(dhp)_2_]–Lyz (14649 Da) plus {[V^IV^O(dhp)(H_2_O)] + [V^IV^O(dhp)_2_]}–Lyz (14870 Da), while when the ratio was 5:1 the peaks of 2[V^IV^O(dhp)_2_]–Lyz (14993 Da) were also identified (Figure 7). With increasing the VC/Lyz ratio, the relative amount of [V^IV^O(dhp)_2_]–Lyz increases compared to [V^IV^O(dhp)(H_2_O)]–Lyz. It must be noticed that in the moieties V^IV^O(dhp)_2_ and V^IV^O(dhp)(H_2_O)^+^, one (equatorial) and two coordination sites (equatorial and axial) are available for coordination to the protein.

Comparing the results of Hdhp with those obtained with the structurally similar ligand Hma, we found that in the first case adducts with the 1:2 fragment V^IV^O(dhp)_2_ are mainly observed, while for maltolate the 1:1 moiety, V^IV^O(ma)^+^, binds preferentially to lysozyme.

### Interaction of [V^IV^O(acac)_2_] with Lysozyme

Spectroscopic and potentiometric studies indicate that acetylacetonate forms with V^IV^O^2+^ the species with 1:1 and 1:2 composition,^30^ at which the stoichiometries [V^IV^O(acac)(H_2_O)_2_]^+^ and [V^IV^O(acac)_2_], with square pyramidal geometry, are assigned.^31^

The ESI-MS spectrum obtained after dissolution in ultrapure water of the solid complex [V^IV^O(acac)_2_] was recorded in this study (Figure S7). At *m*/*z* 101.06 and *m*/*z* 123.04, the protonated form of the ligand [Hacac+H]^+^ and the sodium adduct [Hacac+Na]^+^ are observed. The spectrum shows also the peaks at *m*/*z* = 266.04 and *m*/*z* = 288.02, which were attributed to [V^IV^O(acac)_2_+H]^+^ and [V^IV^O(acac)_2_+Na]^+^, respectively. The comparison between the experimental and calculated isotopic pattern for the peak of the [V^IV^O(acac)_2_+Na]^+^ ion is shown as an example in Figure S8. In the negative-ion mode spectrum, the signal of the species [V^IV^O(acac)_2_–H]^−^ at *m*/*z* 264.02 was observed with a low intensity. All the species detected are listed in Table S4.

**Figure 8:**
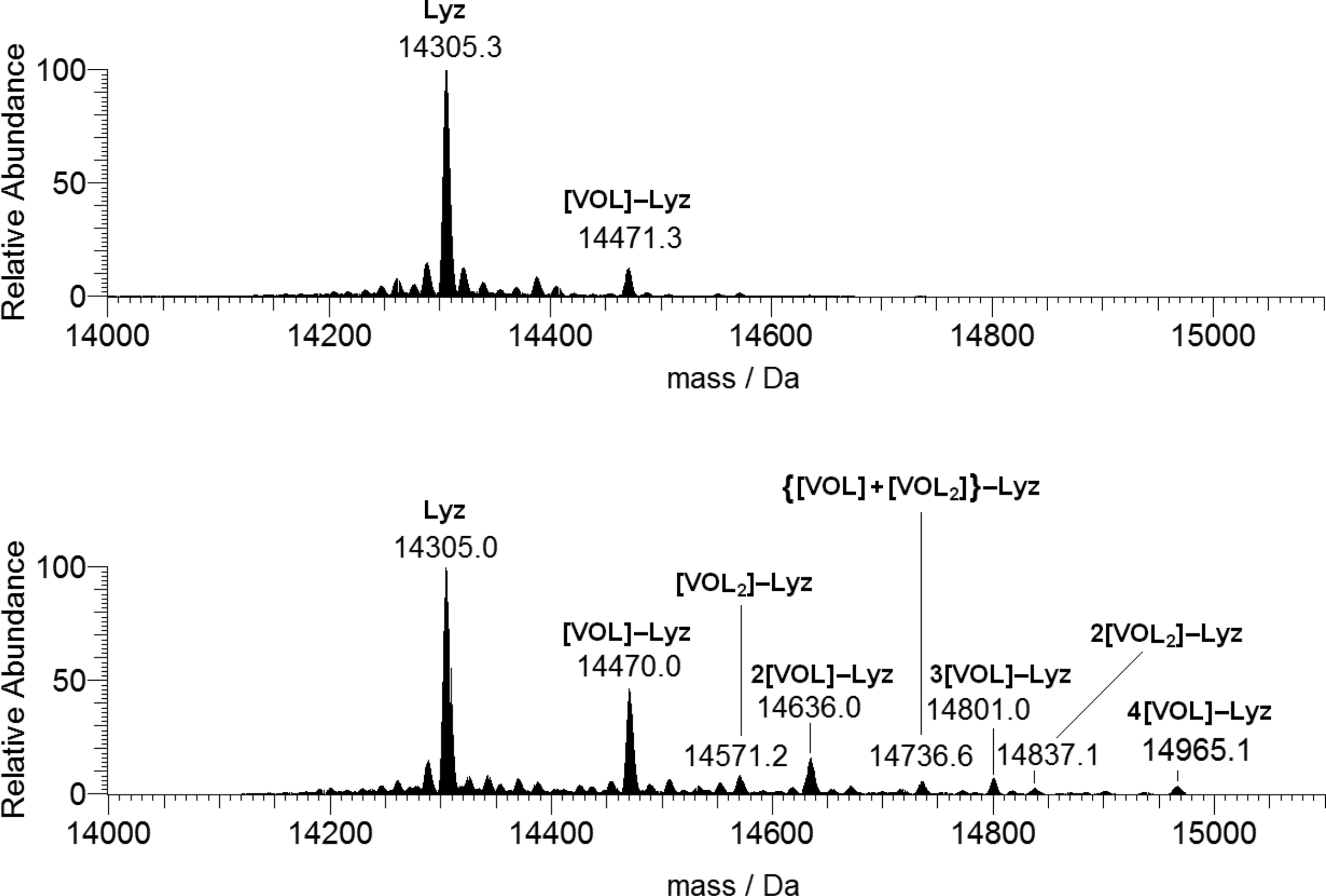
Deconvoluted ESI-MS spectra recorded on the system containing [V^IV^O(acac)_2_] and lysozyme with a molar ratio of 5:1 and a protein concentration of 5 μM (top) and 50 μM (bottom). L indicates the acetylacetonato ligand.

The ESI-MS spectrum recorded on the system [V^IV^O(acac)_2_]/Lyz with a ratio of 5:1 and protein concentration of 5 μM shows the signal of the free protein at 14 305 Da and only a weak signal attributable to the adduct [V^IV^O(acac)]–Lyz (Figure 8, top). For this species the equatorial interaction with one or two protein residues (plus a weak axial binding) is possible. When a higher concentration is used (50 μM), the spectrum shows a series of peaks belonging to [V^IV^O(acac)]–Lyz (14 470 Da), 2[V^IV^O(acac)]–Lyz (14 636 Da), 3[V^IV^O(acac)]–Lyz (14 801 Da), and 4[V^IV^O(acac)]–Lyz (14 965 Da), while the weak signals at 14 571 and 14 837 Da can be attributed to [V^IV^O(acac)_2_]–Lyz and 2[V^IV^O(acac)_2_]–Lyz (Figure 8, bottom). Therefore, the increase of the vanadium concentration favors the interaction of the 1:2 complex [V^IV^O(acac)_2_] with lysozyme, even though—as discussed above for maltolate—the binding of the 1:1 species V^IV^O(acac)^+^ is favored over [V^IV^O(acac)_2_] (see Table 1). Concerning the interaction between [V^IV^O(acac)_2_] with lysozyme, no equatorial sites are available, and so only two possibilities exist: a weak axial binding through an amino acid residue or a noncovalent interaction on the protein surface.

### Interaction of Amavadin with Lysozyme

Amavadin, isolated in 1972 by Kneifel and Bayer,^32^ is an anionic nonoxido V^IV^ compound accumulated in three species of mushrooms of the genus *Amanita*: *A. muscaria*, *A. regalis* and *A. velatipes*. In *A. muscaria* the vanadium content exceeds 400 times the value normally detected in other species of the same genus and is independent of the vanadium concentration in the soil.^4,17^ It is formed by the ligand *N*-hydroxyimino-2,2′-diisopropionic acid, (*S*,*S*)-H_3_hidpa (Scheme 1), in its triply deprotonated form hidpa(3−). The XRD structure of the calcium salt of amavadin, [Ca(H_2_O)_5_][V^IV^((*S*,*S*)-hidpa)_2_]·2H_2_O, shows an eight-coordination around V^IV^, involving two η^2^-N,O^–^ groups and four monodentate carboxylates.^17^

In this study, the negative-ion mode ESI-MS spectrum was recorded dissolving the solid complex [Ca(H_2_O)_5_][V^IV^(hidpa)_2_]·2H_2_O in ultrapure water. It shows the presence of a peak at *m*/*z* = 399.02 attributed to the anionic species [V^V^(hidpa)_2_]^−^. The detection of the V^V^ species is due to the presence in solution of [V^IV^(hidpa)_2_]^2–^ and can be attributed—as mentioned above—to the oxidation of the complex in source or in solution.^25^ The comparison between the experimental and calculated isotopic pattern confirms the presence of this species (Figure S9).

**Figure 9:**
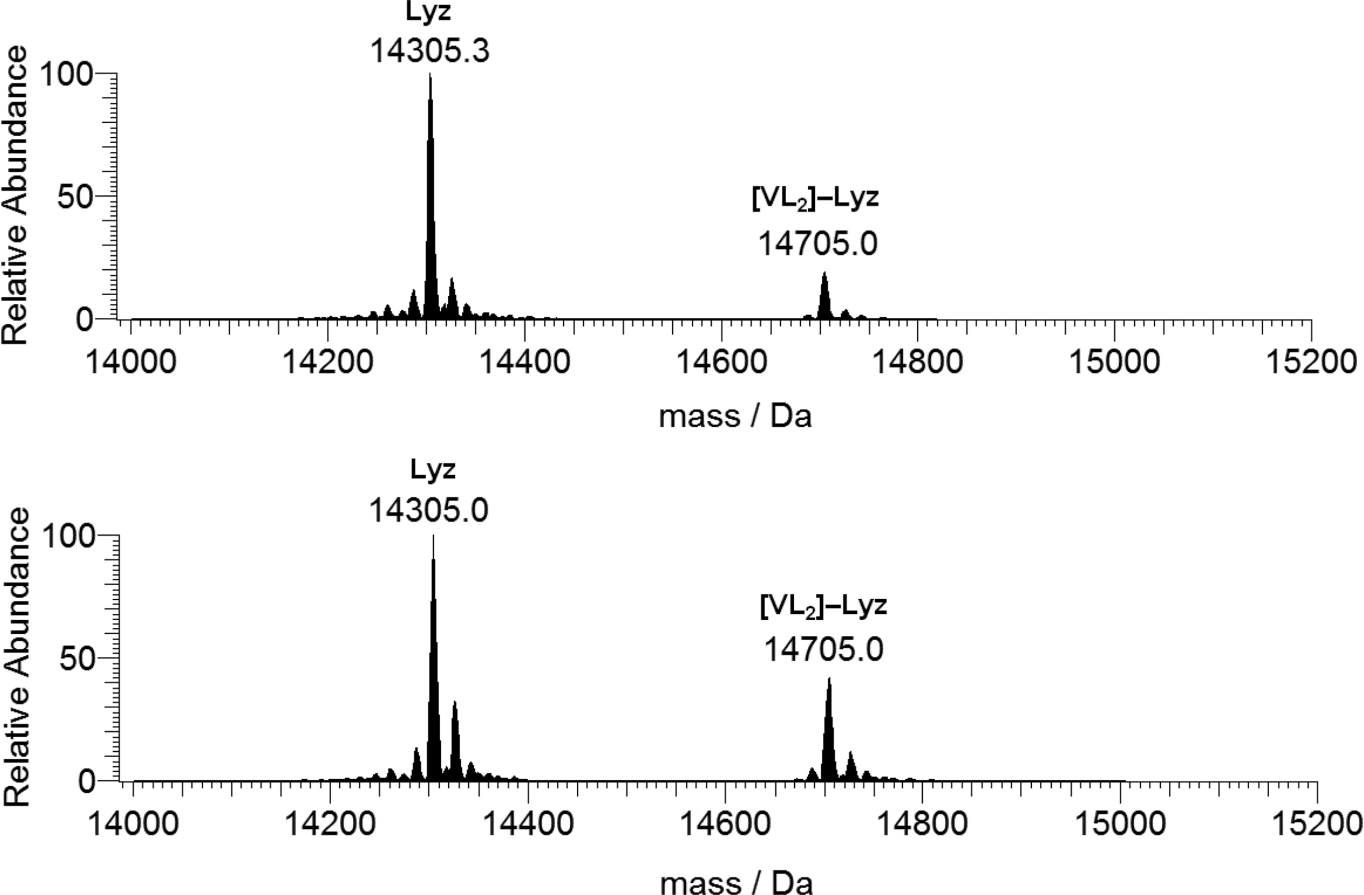
Deconvoluted ESI-MS spectra recorded on the system containing [V^IV^(hidpa)_2_]^2–^ and lysozyme with a molar ratio of 5:1 and a protein concentration of 5 μM (top) and 50 μM (bottom). L indicates the *N*-hydroxyimino-2,2′-diisopropionato ligand.

The ESI-MS spectrum of the system [V^IV^(hidpa)_2_]^2–^/Lyz 5:1 was recorded (Figure 9) and, together with the signal of the free protein, only a peak attributable to the adduct [V(hidpa)_2_]–Lyz was observed (14706.8 Da), whose intensity increases with the vanadium concentration. Considering that several protonation states exist for the protein, it is not possible to determine if the oxidation state of vanadium in the adduct is +IV or +V. On the basis of the studies in the literature, one would expect that it is the V^IV^ complex, because the oxidation to V^V^ is not favored.^17b^

### Interaction of [V^IV^O(pic)_2_(H_2_O)] with Myoglobin

Myoglobin (Mb) is a small protein, found in skeletal muscles and in the heart, where it stores molecular oxygen. It constitutes up to 5–10% of all the cytoplasmic proteins found in these muscle cells and consists of a polypeptide of 153 amino acid residues and a heme group.^33,34^ Under physiological conditions, 70% of the Mb backbone is folded into eight alpha helices (named A–H), the folding leading to a tight globular structure with a cleft for the heme group at helices C, E, and F.^35^

**Figure 10:**
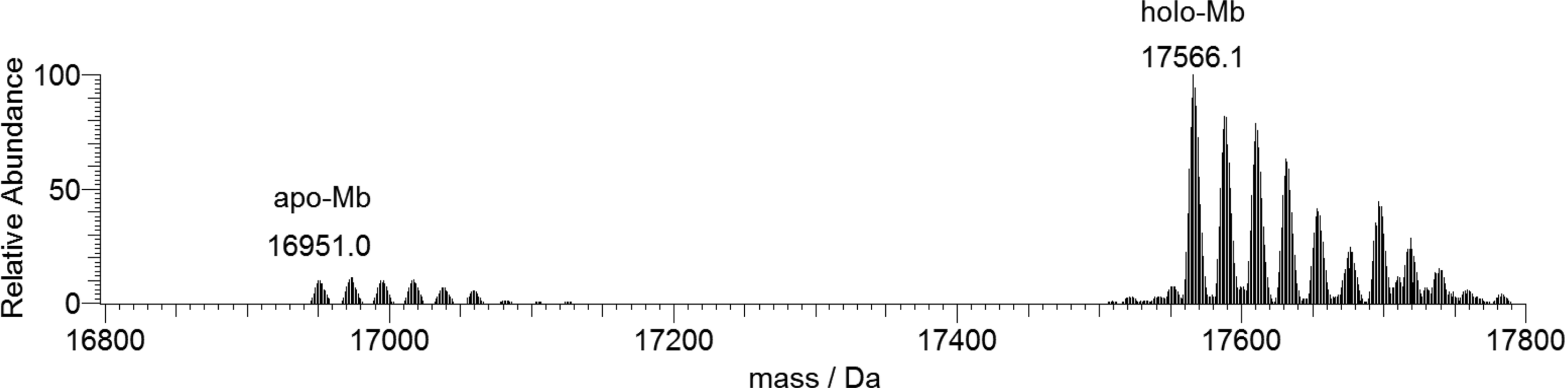
Deconvoluted ESI-MS spectrum of myoglobin (concentration 5 μM).

The ESI-MS spectrum of myoglobin dissolved in ultrapure water (Figure S10) shows a series of peaks which correspond to different charge states (from +6 to +13). In the deconvoluted spectrum, two series of peaks can be recognized with an experimental mass of 16 951 and 17 566 Da, respectively, and a predominance of the second one. The mass difference (615 Da) suggests that they belong to the apo (without heme) and holo forms of the protein, with the latter maintaining the binding with the heme group. It is known that in myoglobin heme is bound to the protein chain by the covalent binding with the proximal histidine (His93) and that the stability of this coordinative bond may be heavily affected by experimental and instrumental conditions.^36^ In particular, the heme group can dissociate at low pH (2–3)—due to protonation of the histidine residues—and strong ionization conditions, while it is preserved at high pH and mild electrospray source conditions. The results obtained in this study are consistent with those reported in the literature^36^ and suggest that, under our experimental conditions, the prosthetic group remains mainly bound to the protein. Analyzing the signal of holo-Mb in Figure 10, it can be noticed that the most intense peak at 17 566 Da is revealed with a series of other signals due to the adducts with a certain number of Na^+^ ions (23 Da).

The presence of the free heme group was confirmed by the detection of a signal at *m*/*z* 616.2 in the raw spectrum of the protein. In Figure S11, a comparison between the experimental and calculated isotopic patterns attributable to [Fe^III^ heme]^+^ is shown; in agreement with the results in the literature, the oxidation state of iron in the heme group released from Mb is +III.^37^

The interaction of [V^IV^O(pic)_2_(H_2_O)] with myoglobin was studied by ESI-MS with the same ratio and concentration used for the previous measurements with lysozyme. In the raw spectrum, the signals at *m*/*z* values higher than the peaks of free protein can be attributed to [V^IV^O(pic)]–Mb and [V^IV^O(pic)_2_]–Mb adducts (Figure S12). The deconvoluted spectra with a protein concentration of 5 μM are shown in Figure S13. The mass difference between the peaks of the adducts and those of free protein suggest that *n*[V^IV^O(pic)_2_]–Mb (*n* = 1–3) and {[V^IV^O(pic)] + *n*[V^IV^O(pic)_2_]}–Mb (*n* = 0–2) are formed (Table 2). This indicates that, under these experimental conditions, only two or three vanadium species are bound to the protein.

**Table 2:** Main Adducts Formed by V^IV^O(pic)_2_(H_2_O) with Myoglobin and Ubiquitin Observed by ESI-MS

protein	mass (Da)	adducts
Mb	17864.0	[V^IV^O(pic)]–Mb
	17986.0	[V^IV^O(pic)_2_]–Mb
	18174.0	{[V^IV^O(pic)] + [V^IV^O(pic)_2_]}–Mb
	18298.0	2[V^IV^O(pic)_2_]–Mb
	18485.0	{[V^IV^O(pic)] + 2[V^IV^O(pic)_2_]}–Mb
	18609.0	3[V^IV^O(pic)_2_]–Mb
	18799.0	{[V^IV^O(pic)] + 3[V^IV^O(pic)_2_]}–Mb
	18920.0	4[V^IV^O(pic)_2_]–Mb
	19109.0	{[V^IV^O(pic)] + 4[V^IV^O(pic)_2_]}–Mb
	19231.0	5[V^IV^O(pic)_2_]–Mb
Ub	8752.6	[V^IV^O(pic)]–Ub
	8875.6	[V^IV^O(pic)_2_]–Ub
	9063.6	{[V^IV^O(pic)] + [V^IV^O(pic)_2_]}–Ub
	9186.6	2[V^IV^O(pic)_2_]–Ub
	9374.6	{[V^IV^O(pic)] + 2[V^IV^O(pic)_2_]}–Ub
	9497.6	3[V^IV^O(pic)_2_]–Ub
	9685.5	{[V^IV^O(pic)] + 3[V^IV^O(pic)_2_]}–Ub
	9809.6	4[V^IV^O(pic)_2_]–Ub

**Figure 11:**
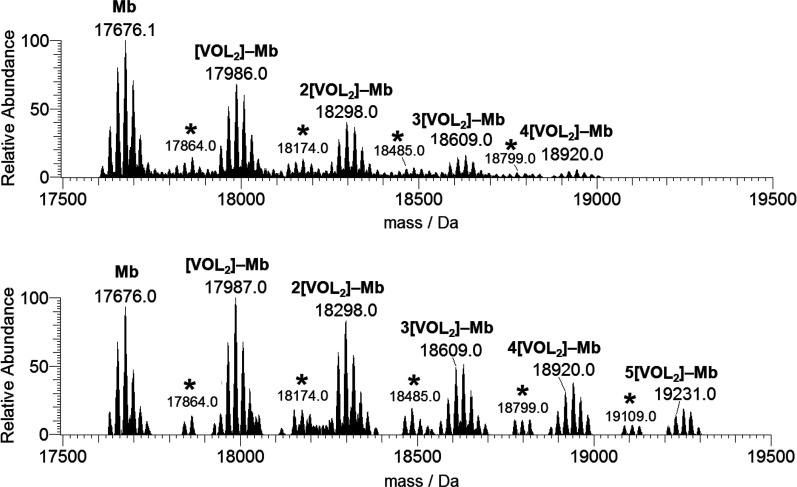
Deconvoluted ESI-MS spectra recorded on the system containing [V^IV^O(pic)_2_(H_2_O)] and myoglobin (50 μM): molar ratios 3:1 (top) and 5:1 (bottom). L indicates the picolinato ligand. With the asterisks, the peaks of the adducts {[V^IV^OL] + *n*[V^IV^OL_2_]}–Mb with *n* = 0–3 (ratio 3:1) and *n* = 0–4 (ratio 5:1) are denoted.

If the spectra are recorded with a protein concentration of 50 μM (Figure 11), the number of V^IV^O(pic)_2_ fragments bound to the protein raises up to 4 when the 3:1 ratio is used and up to 5 with a 5:1 ratio, and a general increase of the relative abundance of the metal–protein adducts is revealed. It can be noticed that at 50 μM the peaks of {[V^IV^O(pic)] + *n*[V^IV^O(pic)_2_]}–Mb are also observed, even though with an intensity lower than that of [V^IV^O(pic)_2_]–Mb (cfr. Figures 11 and S13).

### Interaction of [V^IV^O(pic)_2_(H_2_O)] with Ubiquitin

Ubiquitin is a small regulatory protein constituted of a polypeptide chain of 76 amino acid residues with a molecular weight of 8.5 kDa. It contains a limited number of potential binding sites for metals, among them the N-terminal methionine (Met1), one histidine residue (His68), and some carboxylate O-donor groups.^10c^ Ub is involved in the post-transductional signal called ubiquination, which has a regulatory role in the cellular processes. Besides its functions in biological systems, ubiquitin is used as a model protein in mass spectrometry since it is commercially available with high purity, well characterized, and allows to obtain accurate data.^10c^ The interaction with some metal compounds with antitumoral activity (such as cisplatin and Ru-based complexes) was already studied by MS.^12c,38^

**Figure 12:**
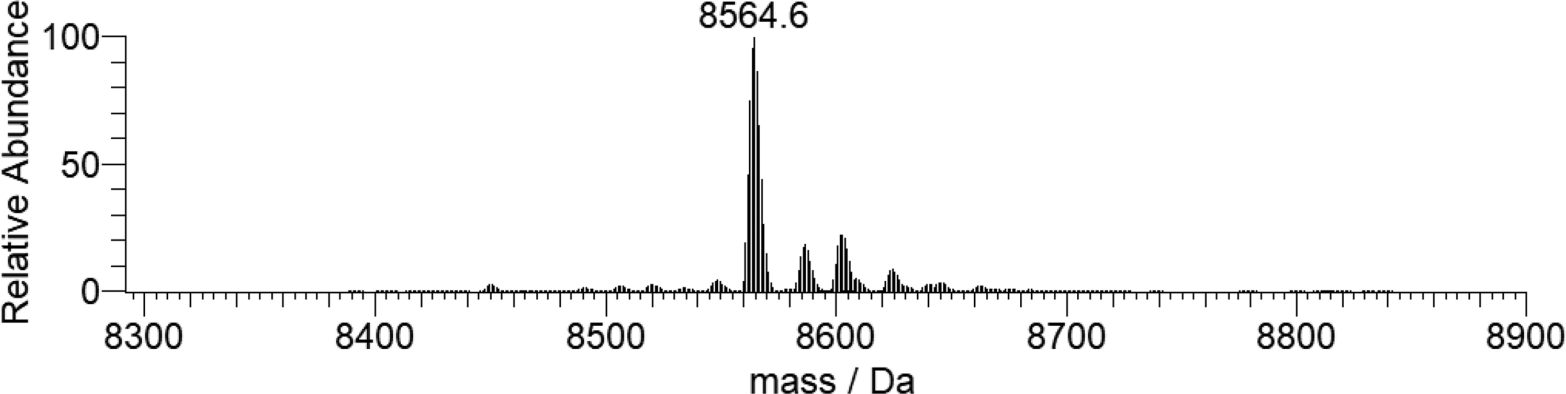
Deconvoluted ESI-MS spectrum of ubiquitin (concentration 5 μM).

The ESI-MS spectrum of the free protein dissolved in ultrapure water at a concentration of 5 μM (Figure S14) was recorded in this study and shows a series of peaks corresponding to the different charge states of ubiquitin (from +5 to +10). The deconvoluted spectrum reported in Figure 12 shows that the mass of the protein is 8564.6 Da, in accordance with the literature data.^12c,38^ In addition to the major peak, a series of other signals due to the adducts with ubiquitous ions, such as Na^+^ (23 Da) and K^+^ (39 Da), are present.

**Figure 13:**
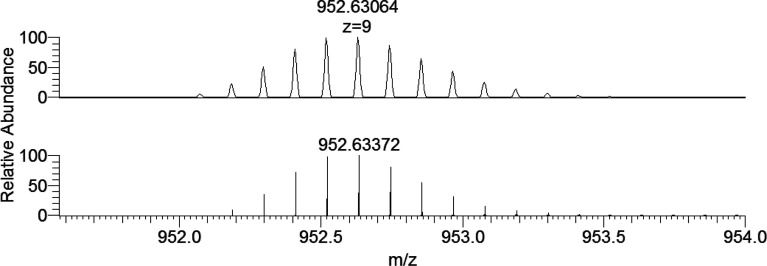
Experimental (top) and calculated (bottom) isotopic pattern for the peak revealed in the ESI-MS spectrum of ubiquitin, C_378_H_639_N_105_O_118_S (i.e., [Ub+9H^+^]) at *m*/*z* 952.63 with *z* = 9.

With ubiquitin, the isotopic pattern of its charged states was simulated, allowing us to determine the exact formula. As an example, in Figure 13 the comparison of the experimental and calculated isotopic pattern for the peak at *m*/*z* 952.63 with *z* = 9 is reported. The empirical formula of the neutral protein is C_378_H_630_N_105_O_118_S, in agreement with the previous studies.^39^

**Figure 14:**
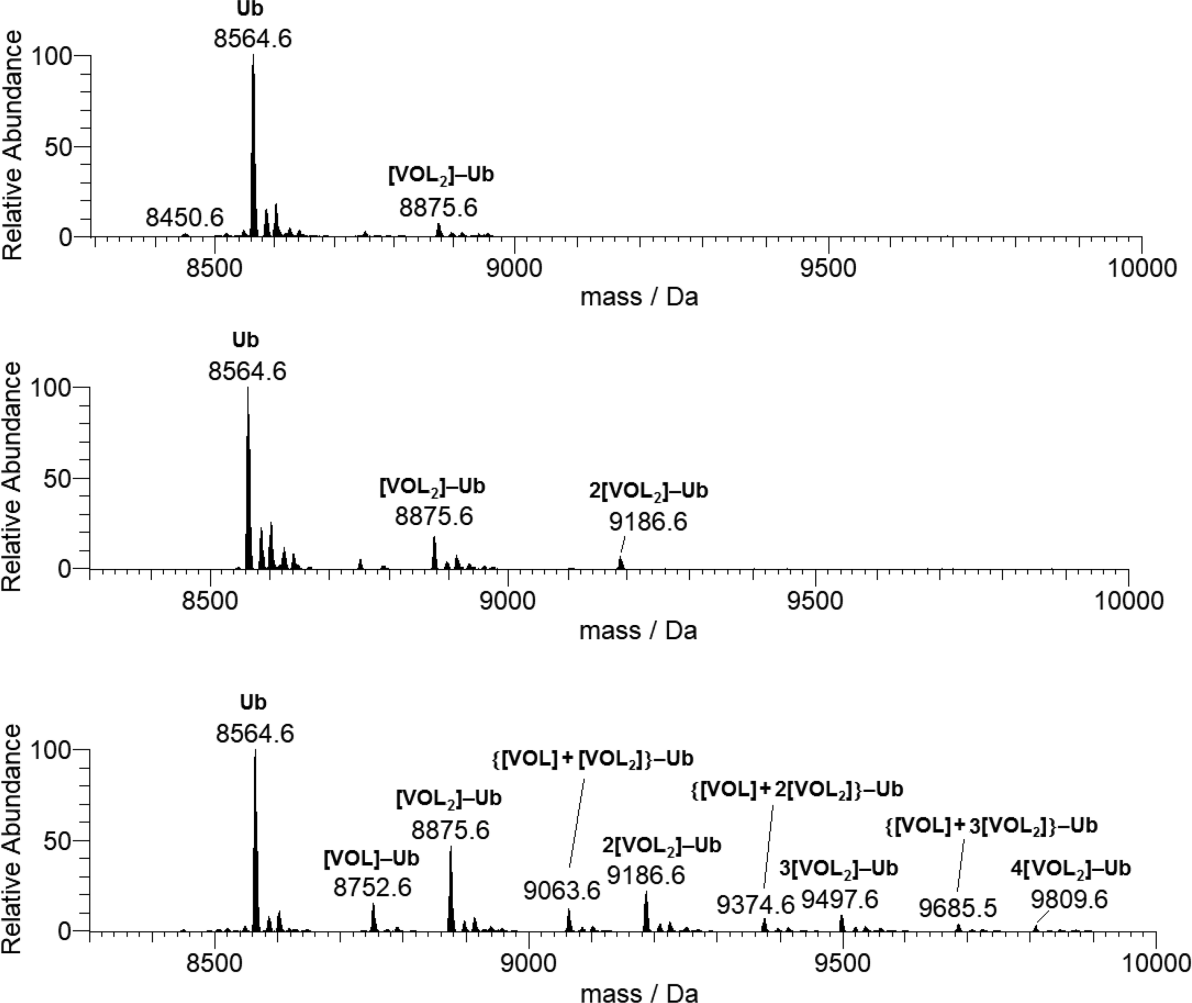
Deconvoluted ESI-MS spectra recorded on the system containing [V^IV^O(pic)_2_(H_2_O)] and ubiquitin (5 μM): molar ratios 3:1 (top), 5:1 (center), and 10:1 (bottom). L indicates the picolinato ligand.

The ESI-MS spectra were recorded on the system [V^IV^O(pic)_2_(H_2_O)]/Ub with different protein concentrations (5 and 50 μM) and metal-to-protein molar ratios (3:1, 5:1, and 10:1). When the signals in the deconvoluted spectra are analyzed, it can be noticed that, in comparison with the free protein (with peak centered at 8564.6 Da, see Figure 12), signals at higher mass appear, suggesting the formation of adducts VC–ubiquitin (Figure 14). At ratio 10:1, the major adducts revealed are *n*[V^IV^O(pic)_2_]–Ub with up to four *cis*-V^IV^O(pic)_2_ fragments bound to the protein; the other peaks correspond to {[V^IV^O(pic)] + *n*[V^IV^O(pic)_2_]}–Ub (*n* = 0–3), similarly to what was observed with lysozyme and myoglobin (Tables 1 and 2). It must be noticed that the intensity of the signals attributed to {[V^IV^O(pic)] + *n*[V^IV^O(pic)_2_]}–Ub is lower than those of *n*[V^IV^O(pic)_2_]–Ub.

To confirm the formation of the adducts, the simulation of the experimental isotopic pattern was carried out for the species formed by V^IV^O(pic)_2_ with Ub obtaining an excellent agreement with the experimental data (Figure S15); it was found that the adduct with molecular formula C_390_H_638_N_107_O_123_SV (*m*/*z* = 1110.46, z = 8) corresponds to [V^IV^O(pic)_2_]–Ub.

Finally, in the spectra recorded with an ubiquitin concentration of 50 μM, the maximum number of fragments bound to the protein increases up to 2 with the ratio 3:1 and up to 3 with the ratio 5:1 (Figure S16). Moreover, at a ratio 5:1 also the adduct {[V^IV^O(pic)] + [V^IV^O(pic)_2_]}–Ub, not observed with the same ratio when Ub concentration is 5 μM, is detected (Figure S16). Therefore, according to the data in the literature,^36a^ it can be concluded that with increasing the concentration of the species, the VC–protein interactions become more favored. All the adducts revealed in the system containing [V^IV^O(pic)_2_(H_2_O)] and ubiquitin are listed in Table 2.

### Rationalization of the ESI-MS Data

Analyzing the data presented in the previous sections, it can be observed that—depending on the molar ratio, vanadium concentration, and type of V^IV^ complex—the number and identity of the adducts revealed change significantly.

An important difference between the four systems containing V^IV^O^2+^ complexes and lysozyme is that for Hpic and Hdhp the formation of adducts with the 1:2 complex, *n*[V^IV^OL_2_]–Lyz, is favored, while for Hma and Hacac the adducts *n*[V^IV^OL]–Lyz are revealed (see Figures 4–8). The reason for this apparent anomaly can be found examining the different distribution of the V^IV^O^2+^ species formed in aqueous solutions in the four systems under the experimental conditions used for recording the ESI-MS spectra (pH in the range 5–6 and metal concentration between 15 and 150 μM). The results are summarized in Table 3. As it can be noticed, at 15 μM, the formation of V^IV^OL_2_ species is favored with Hpic and Hdhp, while with maltolate and acetylacetonate, V^IV^OL^+^ is the predominant complex in solution. Therefore, in the first two systems, the adducts *n*[V^IV^OL_2_]–Lyz are the major species and, in contrast, with Hma and Hacac *n*[V^IV^OL]–Lyz predominate. Conducting experiments at 150 μM, the relative amount of V^IV^OL_2_ increases compared to V^IV^OL^+^ species; as a consequence, with Hpic and Hdhp the adducts *n*[V^IV^OL_2_]–Lyz continue to dominate the spectra, whereas with Hma and Hacac those with composition {[V^IV^OL] + [V^IV^OL_2_]}–Lyz, in addition to *n*[V^IV^OL]–Lyz, are detected. It can be observed that with picolinate the intensity of the peaks of {[V^IV^O(pic)] + *n*[V^IV^O(pic)_2_]}–protein is considerably lower than the corresponding *n*[V^IV^O(pic)_2_]–protein, which reflects the relative amount of V^IV^O(pic)_2_ compared to V^IV^O(pic)^+^ in the vanadium concentration range 15–150 μM (Table 3). The ability of the ligand to form stable adducts influences the type of species observed by ESI-MS. This is clear when comparing the results obtained with two structurally similar ligands—maltolate and 1,2-dimethyl-3-hydroxy-4(*1H*)-pyridinonate. Specifically, Hma, the weaker ligand, forms *n*[V^IV^OL]–Lyz adducts, and Hdhp, a stronger ligand (for the presence of the endocyclic N–CH_3_ group instead of the O atom in the six-membered ring^18a,29,40^), gives mainly *n*[V^IV^OL_2_]–Lyz species. This agrees well with the distribution data of the V^IV^O^2+^/Hma and V^IV^O^2+^/Hdhp systems at the concentrations used to record the ESI-MS spectra (Table 3). In the overall examination of the data, it must be taken into account that the metal ion can be sequestered (that is completely removed from the ligand L^–^) when the protein affinity for V^IV^O^2+^ is very strong: a clear example is transferrin, which in the apo form has two iron binding sites which can accommodate hard metal ions like the oxidovanadium(IV) ion;^2a,2d,3b,3e,3j,3m^ metal ion abstraction could alter the species distribution of V^IV^O^2+^ and L^–^. In contrast, when strong sites are not available (as in the case of the proteins examined in this work), the preferential interaction is with V^IV^OL^+^ or V^IV^OL_2_ moieties and, for this reason, the data obtained by ESI-MS can be used to evaluate the interaction of the metal species with proteins and stability of the adducts.

**Table 3:** Distribution (%) of the Most Important Species Formed in the Systems Containing V^IV^OL_2_ (L = pic^–^, ma^–^, dhp^–^, acac^–^) at pH 5.5 and with Vanadium Concentration of 50 and 150 μM Used for Recording the ESI-MS Spectra^a^

ligand	V conc.	V^IV^O^2+^	[V^IV^OL]^+^	[V^IV^OL_2_]	[V^IV^OL_2_H_–1_]^−^	[(V^IV^O)_2_OH)_5_]^−^	[(V^IV^O)(OH)_3_]^−^
pic^–^	15 μM	1.8	39.2	53.2	1.8	0.3	0.1
	150 μM	0.2	14.4	78.5	2.6	0.0	0.0
ma^–^	15 μM	7.3	51.9	30.2	0.0	4.9	0.2
	150 μM	0.9	26.2	65.4	0.0	0.7	0.3
dhp^–^	15 μM	0.1	15.1	84.7	0.0	0.0	0.0
	150 μM	0.0	5.1	94.8	0.0	0.0	0.0
acac^–^	15 μM	11.2	54.8	17.3	b	11.9	0.4
	150 μM	1.8	38.9	55.6	b	2.9	0.1

a Calculated using the stability data of the species reported in refs.^22b,29,30,41^

b Species not formed in the system with acac.

**Figure 15:**
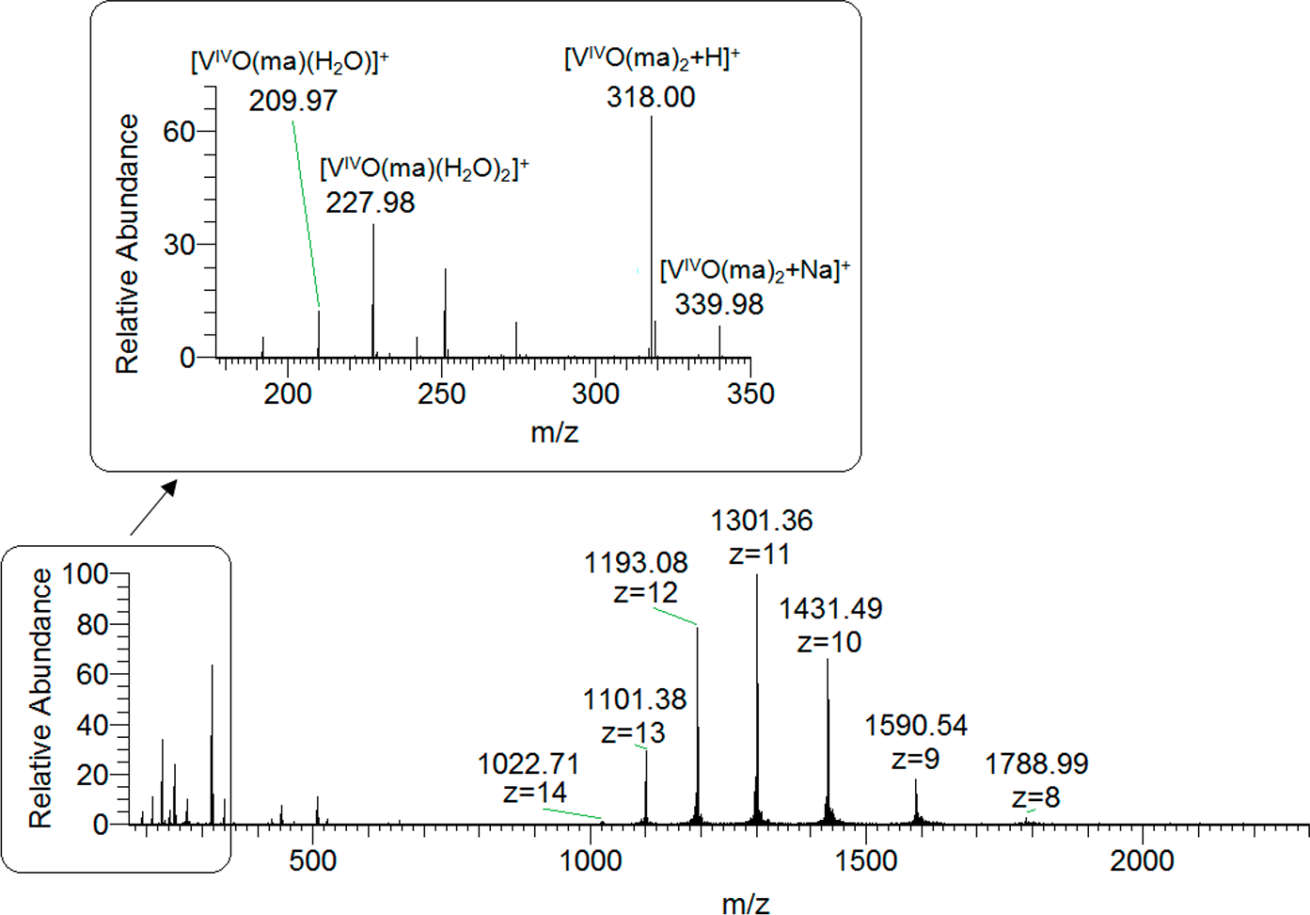
ESI-MS spectrum recorded on the system containing [V^IV^O(ma)_2_] (150 μM) and lysozyme (50 μM). The region in the *m*/*z* range 180–350 is presented in the inset.

The validity of the discussion can be tested analyzing the low *m*/*z* regions of the raw ESI-MS spectra. This is done for the system V^IV^O(ma)_2_/Lyz and is shown in Figure 15 (ratio 3:1 and vanadium concentration 5 μM). Whereas at a high *m*/*z* the signals of the (metal species)–protein adducts are well resolved, at *m*/*z* lower than 350 the peaks of the free complexes are revealed. This proves that an equilibrium between the free complexes and metal adducts with the protein exists according to the reaction VO(ma)_*x*_ + protein ⇄ [VO(ma)_*x*_]–protein, with *x* = 1–2. In the region with *m*/*z* 180–350, besides [V^IV^O(ma)_2_+H]^+^ and [V^IV^O(ma)_2_+Na]^+^ (*m*/*z* = 318.0 and 340.0, respectively), the signals of [V^IV^O(ma)(H_2_O)]^+^ and [V^IV^O(ma)(H_2_O)_2_]^+^ are detected (*m*/*z* = 210.0 and 228.0). Even though the intensity of the MS signal does not accurately report on the concentration of the species in solution, the relative intensity indicates that the 1:2 complex is favored, according to the data of Table 3. In contrast, the concentration of the hydrolytic species [(V^IV^O)_2_(OH)_5_]^−^ and [(V^IV^O)(OH)_3_]^−^ is so small that, under these experimental conditions, they cannot be observed. The simulations with the software Thermo Xcalibur of the isotopic patterns of [V^IV^O(ma)(H_2_O)]^+^, [V^IV^O(ma)(H_2_O)_2_]^+^, [V^IV^O(ma)_2_+H]^+^, and [V^IV^O(ma)_2_+Na]^+^ are shown in Figures S17–S20.

In the other systems, a similar situation is observed. Notably, the ratio between the signal intensity of the 1:2 complexes to that of the 1:1 ones increases from acac^–^ to pic^–^ and to dhp^–^, in line with what is expected on the basis of the thermodynamic data (Table 3). The regions with a low *m*/*z* for these systems are reported in Figure S21, while the simulations of the peaks for 1:1 and 1:2 complexes are in Figures S22–S33. Another intriguing hypothesis—that could be investigated in a future study—is that one of the species [V^IV^O(L)(H_2_O)]^+^ or [V^IV^O(L)(H_2_O)_2_]^+^ could be interpreted as the monohydroxide derivative [V^IV^O(L)(OH)+H]^+^ or [V^IV^O(L)(OH)(H_2_O)+H]^+^; these latter, in their turn, would be the precursors of the μ-hydroxide dimer [(V^IV^O)_2_(L)_2_(OH)_2_], detected by potentiometry in the systems with pic^–^, ma^–^, and dhp^–^ and neglected—in a first approximation—with acac^–^.^22b,29,30,41^

### Pharmacological and Biological Implications

The data obtained allow us to advance some hypotheses on the metal–protein adducts formed by V^IV^O^2+^ complexes with concentrations used in this study (between 15 and 150 μM, which is close to that found under the physiological conditions^3m^). The compounds can be divided into two classes: the first class comprises VCs formed by strong ligands (for example, picolinate and 1,2-dimethyl-3-hydroxy-4(1*H*)-pyridinonate) which give adducts with a composition of *n*[V^IV^OL_2_]–protein, the second class includes VCs with intermediate strength ligands (such as maltolate and acetylacetonate) that form adducts *n*[V^IV^OL]–protein.

**Scheme 2:**
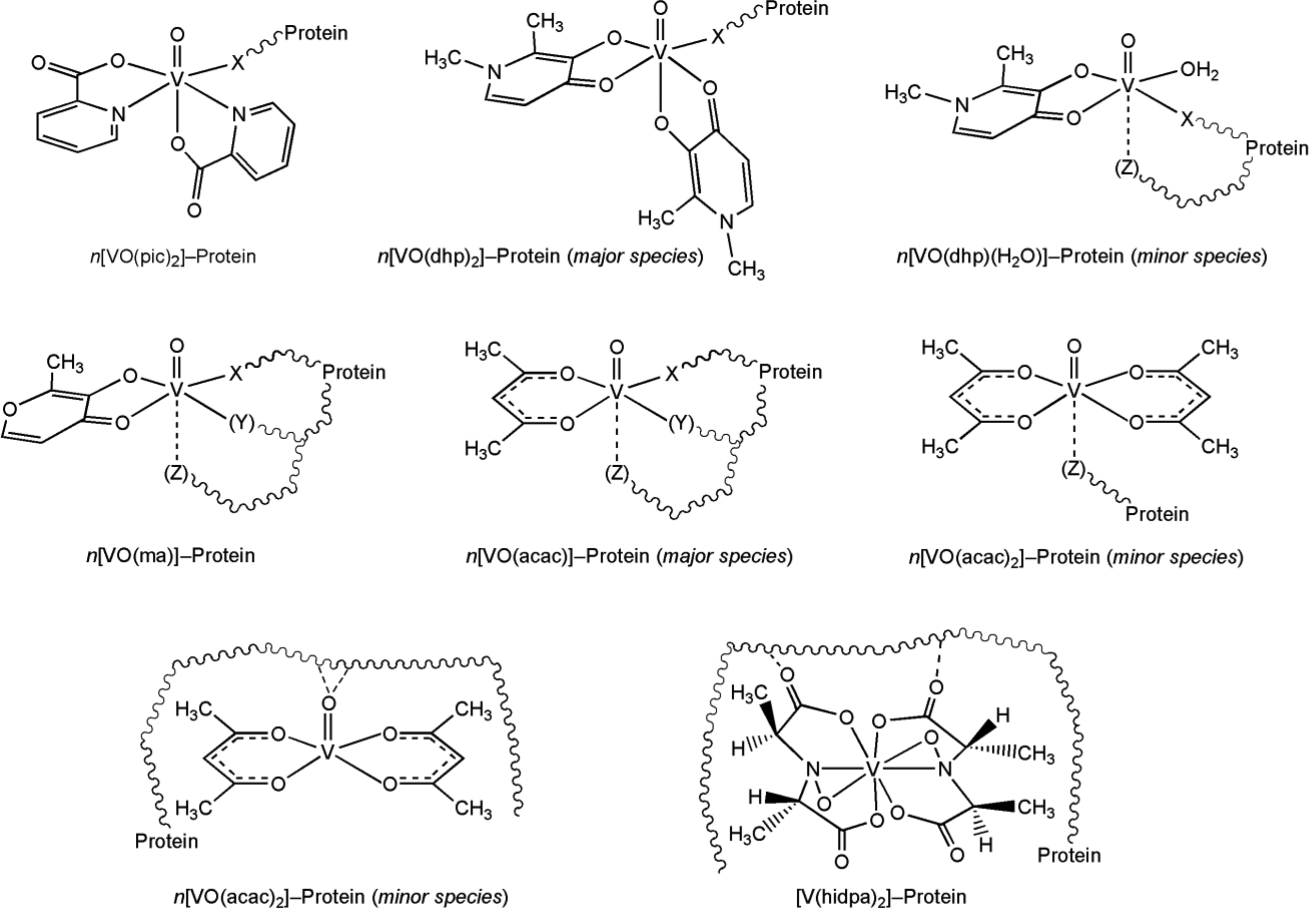
Adducts Formed at the Physiological Vanadium Concentration by [V^IV^O(pic)_2_(H_2_O)], [V^IV^O(ma)_2_], [V^IV^O(dhp)_2_], [V^IV^O(acac)_2_], and [V^IV^(hidpa)_2_]^2–^, When more than one adduct is formed in aqueous solution, it is indicated with *minor* or *major species*. In round parentheses, the less probable binding of an axial residue *Z* is denoted. The charges of the ligands and V=O ion (2+) are omitted for clarity.

In general, for Hpic and Hdhp adducts, the protein binds with an accessible protein residue indicated with an X in Scheme 2 in the fourth equatorial position. Specifically, the structure of the adduct between *cis*-V^IV^O(pic)_2_ and Lyz has been determined by X-ray crystallography and computational methods, and the Asp52 residue of lysozyme was found to replace the equatorial water ligand of V^IV^O(pic)_2_(H_2_O) forming a distorted octahedral complex.^3h,42^ Glu and His are other residues able to bind V in a monodentate manner. Particularly, accessible residues for the coordination to a *cis*-V^IV^OL_2_ moiety are Glu35 with Lyz,^42^ Glu16, Glu18, Asp21, and His68 for Ub,^43^ while for Mb they are mainly histidines (His81, His113, His116, His199).^44^ With Hdhp, an identical behavior is expected with the formation of *n*[V^IV^O(dhp)_2_]–protein species with the equatorial binding of Asp, Glu, and His; moreover, the formation of a minor adduct with the composition [V^IV^O(dhp)(H_2_O)]–protein was observed, with the coordination of the X residue in the equatorial plane and, eventually, Z in the axial position.

With ma^–^ and acac^–^, the adducts formed have the general formula *n*[V^IV^OL]–protein. A considerable number of adducts are possible and should be considered. Since there are two free *cis* equatorial positions (plus the axial site) in the V^IV^OL^+^ moiety, the protein binding mode could be mono-, bi-, or tridentate (X, Y, plus a further axial Z, Scheme 2). The bidentate or tridentate binding mode of the protein depends on the protein conformation and on the eventual presence of two or three neighboring residues able to occupy contemporaneously three *facial* positions of the octahedral vanadium geometry. This eventuality is rare in small proteins such as lysozyme but could be more common in larger proteins such as transferrin or albumin. The candidate residues able to coordinate the V center are Asp, Glu, and His, with the possible assistance of Ser, Thr, Tyr, Cys, or backbone-CO if they are in the right position. With the acac^–^ ligand, adducts with formula *n*[V^IV^O(acac)_2_]–protein are also observed. Since [V^IV^O(acac)_2_] is square pyramidal and no equatorial sites are available, in such species the proteins could interact weakly with a residue Z in the axial position or, alternatively, with weak noncovalent interactions with residues on the protein surface (see Scheme 2 and ref (45)). If the interaction is strong enough, these adducts can be revealed by an ESI-MS technique.

If the VC is very stable, such as amavadin, and no coordination sites in the complex are available, only noncovalent interactions could occur (Scheme 2). The strength of these interactions depends on the number of polar groups in the VC able to form hydrogen bonds, van der Waals, or hydrophobic contacts with the exposed polar groups on the protein surface.^45^

These studies convincingly show that, if a VC reaches the blood, a significant amount of metal–protein adducts exist and the type of interaction can influence its fate in the organism and favor the cellular uptake and binding to the cellular receptor(s). For example, the adducts with transferrin, with the binding of V^IV^O^2+^ to the active sites left free by Fe^3+^ or to the accessible surface donors, can promote a conformational change which would lead to recognition by the cellular receptors in the endocytosis.^46^ Moreover, the binding to the cellular targets can lead to pharmacological activity, as in the inhibition of phosphatases, to which the vanadium antidiabetic activity is attributed.^47^ In this context, the results obtained with the ESI-MS technique could be considered in the design of new potential vanadium therapeutics. First of all, ESI-MS allows the study of biospeciation at the metal concentrations found in the patients treated with potential drugs. Second, the redox reactions in the biological media containing proteins of the three oxidation states, V^III^, V^IV^O, and V^V^O_2_, both in the free form and in the generated adducts, can be followed with the aim to determine the most stable state. Third, ESI-MS can suggest if the metal species exists in the free form or bound to the protein, and which equilibriums in solution are established and which adducts are formed (see Figure 15). Depending on the molecular receptor(s), the features of the organic ligand L^–^ could be modulated to favor formation at physiological concentrations of moieties VOL^+^, that can form stable species with proteins through the binding of two amino acid donors, or VOL_2_, that—in contrast—yields moderately stable adducts if it is in the *cis* arrangement or unstable if the interaction occurs in the axial position (Scheme 2).

## Conclusions

In this study, the first systematic application of the ESI-MS technique on the binding to model proteins, such as lysozyme, myoglobin, and ubiquitin, of four V^IV^OL_2_ complexes—[V^IV^O(pic)_2_(H_2_O)], [V^IV^O(ma)_2_], [V^IV^O(dhp)_2_], and [V^IV^O(acac)_2_], which are reported to normalize elevated glucose levels in diabetic animals and to be effective on several tumor cell lines—is presented. The interaction with lysozyme of the vanadium-containing natural product amavadin is also investigated. The results indicate that ESI-MS can be successfully applied to these systems with reproducible results and gives information on the number and stoichiometry of the species formed in the metal concentration range found in the organisms treated with VCs. In particular, adducts with different compositions were found, *n*[V^IV^OL_2_]–Lyz or *n*[V^IV^OL]–Lyz, where the value of *n* varies with changing L. This experimental evidence allows for ascertaining the biospeciation of a pharmacologically active V^IV^OL_2_ complex with vanadium concentrations in the range 10–100 μM. For example, if V^IV^OL_2_ is stable, as found with picolinate and 1,2-dihydroxy-3-methyl-4(1*H*)-pyridinonate, it survives in solution even at micromolar concentrations and can bind to proteins as 1:2 species. The comparison between the [V^IV^O(pic)_2_(H_2_O)] binding to lysozyme, myoglobin, or ubiquitin indicates a similar interaction, with the *cis*-V^IV^O(pic)_2_ moiety which coordinates to proteins through the free equatorial site. In contrast, if the VC is less stable, as for maltolate and acetylacetonate, it undergoes hydrolysis, and the 1:1 moiety V^IV^OL^+^ interacts with the proteins. It is expected that with weaker ligands, such as 6-methylpicolinate or kojate, the formation of hydrolytic species like [(V^IV^O)_2_(OH)_5_]^−^ and [V^IV^O(OH))_3_]^−^ may occur, with the possible further oxidation to V^V^ inorganic anions. Two important findings are: (i) the number of metal fragments bound to proteins increases with the VC concentration and (ii) the oxidation to V^V^ is possible in the binary systems V^IV^O^2+^–L, but it is prevented or significantly slowed down after the formation of the adducts VC–protein. In fact, in the studied systems, there is no evidence of oxidation of V^IV^ to V^V^.

The results could allow the prediction of the fate of an administered V^IV^OL_2_ potential drug. If the complex is stable, the proteins can coordinate the V^IV^OL_2_ moiety only in a monodentate manner, remembering that the interaction and its strength are related to the geometry assumed in solution, *cis*-octahedral with a free equatorial position (moderately strong binding) or square pyramidal with an available axial site (weak coordinative or noncovalent interaction). If the complex has an intermediate stability, the proteins can interact with the VOL^+^ fragment in a bi- or tridentate fashion, generating stable adducts with the contemporaneous binding of two amino acid side-chains in two adjacent equatorial positions. Finally, when the V^IV^ complex is very stable without available coordination sites, as happens for amavadin, the proteins are not able to interact covalently and only weak noncovalent contacts are predicted. Since the formed adducts have different thermodynamic stability, it is clear that, depending on the properties of the ligand L, a pharmacologically active V^IV^O^2+^ complex would reach the target organs in different forms, each of them being characterized by a different cellular uptake and biological activity. Thus, in relation to the pharmacological activity, not only the solubility and hydrophobicity of the VCs are important but also their ability to interact with proteins, which determines the adducts existing under physiological conditions. Overall, the results further support the fact that the biological properties of VCs must be related with their biospeciation in the organism and that a mixture of species (intact VC, adducts with proteins and/or low molecular mass bioligand, inorganic and hydrolytic ions, this latter eventually generated by redox reactions) could contribute to the pharmacological action,^48^ with the active species depending both on the thermodynamic stability and structural requirement of the administered VC and, eventually, on its redox behavior.

## Supporting Information Available

Tables with identified species in the binary systems (Tables S1–S4); raw ESI-MS spectra recorded on the systems containing [VO(pic)_2_(H_2_O)], [VO(acac)_2_], Mb, [VO(pic)_2_(H_2_O)]/Mb, and Ub (Figures S1, S7, S10, S12, and S14); experimental and simulated isotopic patterns of [V^IV^O(pic)_2_+H]^+^, [V^IV^O(pic)_2_(OH)]^−^/[V^V^O_2_(pic)_2_]^−^, [V^IV^O(ma)_2_+H]^+^, [V^V^O_2_(ma)_2_]^−^, [V^IV^O(dhp)_2_+Na]^+^, [V^IV^O(acac)_2_+Na]^+^, [V^V^(hidpa)_2_]^−^, heme group in myoglobin, [V^IV^O(pic)_2_]–Ub (Figures S2–S6, S8, S9, S11 and S15); deconvoluted ESI-MS spectra recorded on the systems containing [VO(pic)_2_(H_2_O)]/Mb and [VO(pic)_2_(H_2_O)]/Ub (Figures S13 and S16); simulations of the peaks of the free species detected in the systems [V^IV^O(ma)_2_]/Lyz, [V^IV^O(pic)_2_(H_2_O)]/Lyz, [V^IV^O(dhp)_2_]/Lyz, [V^IV^O(acac)_2_]/Lyz (Figures S17–S20 and S22–S33); low *m*/*z* region of the ESI-MS spectra recorded on the systems [V^IV^O(pic)_2_(H_2_O)]/Lyz, [V^IV^O(dhp)_2_]/Lyz, [V^IV^O(acac)_2_]/Lyz (Figure S21); and oxidation process of *cis*-V^IV^O(H_2_O)^2+^ to *cis*-V^V^O_2_^+^ moiety (Scheme S1) (PDF)
