# Supplementary material for: ESI-MS Study of the Interaction of Potential Oxidovanadium(IV) Drugs and Amavadin with Model Proteins
Source: Inorg Chem. 2020 Jun 25;59(14):9739–55. doi: 10.1021/acs.inorgchem.0c00969 (PMC8008395; doi:10.1021/acs.inorgchem.0c00969)
Supplement: Supplementary file 1 — ic0c00969_si_001.pdf [file ic0c00969_si_001.pdf]

SUPPORTING INFORMATION

ESI-MS Study of the Interaction of Potential Oxidovanadium(IV) Drugs and Amavadin with Model Proteins

Valeria Ugone,[§] *Daniele Sanna,*[†] *Giuseppe Sciortino,*[§,#] *Debbie C. Crans,*[¶] *and Eugenio Garribba**[,§]

[§] Dipartimento di Chimica e Farmacia, Università di Sassari, Via Vienna 2, I-07100 Sassari, Italy

[†] Istituto CNR di Chimica Biomolecolare, Trav. La Crucca 3, I-07040 Sassari, Italy

[#] Departament de Química, Universitat Autònoma de Barcelona, 08193 Cerdanyola del Vallés, Barcelona, Spain

[¶] Department of Chemistry, Colorado State University, 1301 Center Avenue, Fort Collins, Colorado, USA

Corresponding author. E-mail: garribba@uniss.it (E.G.).

Table S1. Identified species in the ESI-MS spectra of the system containing $[V^{IV}O(pic)_2(H_2O)]$.

Ion	Composition	Experimental m/z [a]	Calculated m/z [a]	Error (ppm) [b]
$[Hpic+H]^+$	$C_6H_6NO_2$	124.03952	124.03930	-1.8
$[Hpic+Na]^+$	$C_5H_6NO_2Na$	146.02125	146.02125	0.0
$[pic]^-$	$C_6H_4NO_2$	122.02360	122.02475	9.4
$[V^{IV}O(pic)_2+H]^+$	$C_{12}H_9N_2O_5V$	311.99453	311.99456	0.1
$[V^{IV}O(pic)_2+Na]^+$	$C_{12}H_8N_2O_5VNa$	333.97647	333.97651	0.1
$[V^{IV}O(pic)_2(OH)]^-$	$C_{12}H_8N_2O_6V$	326.98299	326.98275	-0.7
$[V^VO_2(pic)_2]^-$	$C_{12}H_9N_2O_6V$	327.99083	327.99057	-0.8

[a] Experimental and calculated m/z values refer to the peak representative of the monoisotopic mass.

[b] Error in ppm respect to the experimental value, calculated as: $10^6 \times$ {[| Calculated (m/z) – Experimental (m/z) |] / Calculated (m/z)}.

Table S2. Identified species in the ESI-MS spectra of the system containing $[V^{IV}O(ma)_2]$.

Ion	Composition	Experimental m/z [a]	Calculated m/z [a]	Error (ppm) [b]
$[Hma+H]^+$	$C_6H_7O_3$	127.03908	127.03897	-0.9
$[ma]^-$	$C_6H_5O_3$	125.0234	125.0244	8.0
$[V^{IV}O(ma)_2+H]^+$	$C_{12}H_{11}O_7V$	317.99328	317.99389	1.9
$[V^{V}O_2(ma)_2]^-$	$C_{12}H_{10}O_8V$	332.98251	332.98208	-1.3

[a] Experimental and calculated m/z values refer to the peak representative of the monoisotopic mass.

[b] Error in ppm respect to the experimental value, calculated as: 10^6 × {[| Calculated (m/z) – Experimental (m/z) |] / Calculated (m/z)}.

Table S3. Identified species in the ESI-MS spectra of the system containing $[V^{IV}O(dhp)_2]$.

Ion	Composition	Experimental m/z [a]	Calculated m/z [a]	Error (ppm) [b]
$[V^{IV}O(dhp)_2+H]^+$	$C_{14}H_{17}N_2O_5V$	344.05698	344.05716	0.5
$[V^{IV}O(dhp)_2+Na]^+$	$C_{14}H_{16}N_2O_5VNa$	366.03958	366.03911	-1.3

[a] Experimental and calculated m/z values refer to the peak representative of the monoisotopic mass.

[b] Error in ppm respect to the experimental value, calculated as: 10^6 × {[| Calculated (m/z) – Experimental (m/z) |] / Calculated (m/z)}.

Table S4. Identified species in the ESI-MS spectra of the system containing $[V^{IV}O(acac)_2]$.

Ion	Composition	Experimental m/z [a]	Calculated m/z [a]	Error (ppm) [b]
$[Hacac+H]^+$	$C_5H_9O_2$	101.06019	101.05971	-4.7
$[Hacac+Na]^+$	$C_5H_8O_2Na$	123.04197	123.04165	-2.6
$[V^{IV}O(acac)_2+H]^+$	$C_{10}H_{15}O_5V$	266.03553	266.03536	-0.6
$[V^{IV}O(acac)_2+Na]^+$	$C_{10}H_{14}O_5VNa$	288.01750	288.01731	-0.7

[a] Experimental and calculated m/z values refer to the peak representative of the monoisotopic mass.

[b] Error in ppm respect to the experimental value, calculated as: $10^6 \times \{[|$ Calculated (m/z) – Experimental (m/z) $|]$ / Calculated (m/z)$\}$.

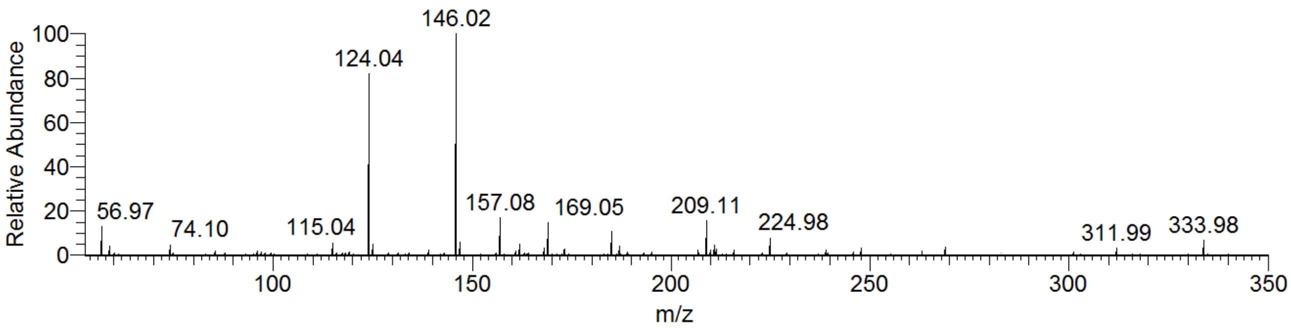

Figure S1. ESI-MS spectrum recorded in positive mode at pH 5.15 on the system containing [$V^{IV}O(pic)_2(H_2O)$] in ultrapure LC-MS water with a V concentration of 5 μM.

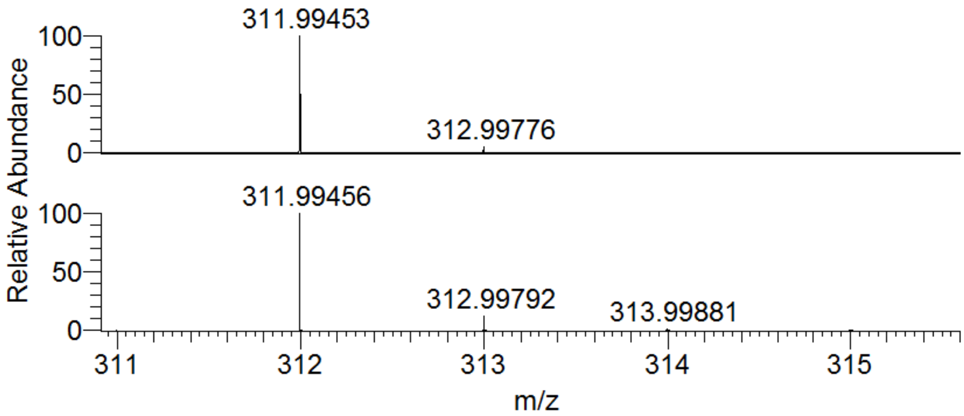

Figure S2. Experimental (top) and calculated (bottom) isotopic pattern of the species $[V^{IV}O(pic)_2+H]^+$ (m/z = 311.99).

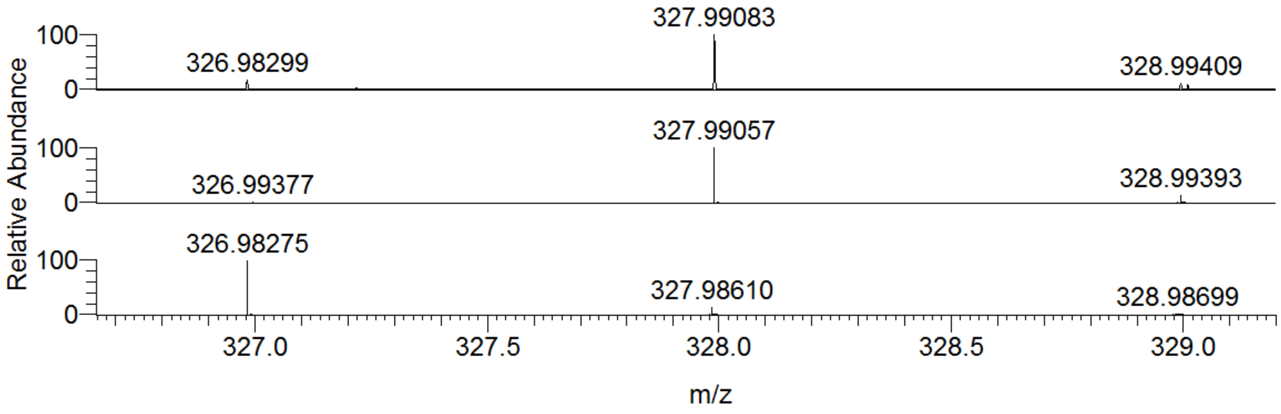

Figure S3. Experimental (top) and calculated isotopic pattern of $[V^{IV}O(pic)_2(OH)]^-$ (centre) and $[V^{V}O_2(pic)_2]^-$ (bottom). m/z = 327.99 for $[V^{IV}O(pic)_2(OH)]^-$ and m/z = 326.98 for $[V^{V}O_2(pic)_2]^-$.

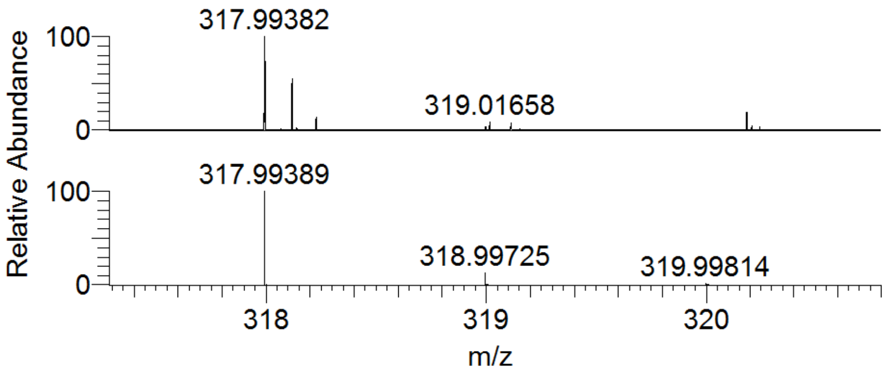

Figure S4. Experimental (top) and calculated (bottom) isotopic pattern of the species $[V^{IV}O(ma)_2+H]^+$ (m/z = 317.99).

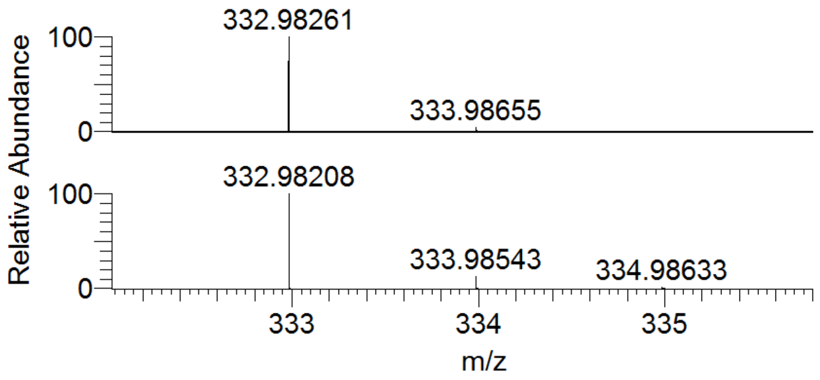

Figure S5. Experimental (top) and calculated (bottom) isotopic pattern of the species $[V^{V}O_2(ma)_2]^{-}$ (m/z = 332.98).

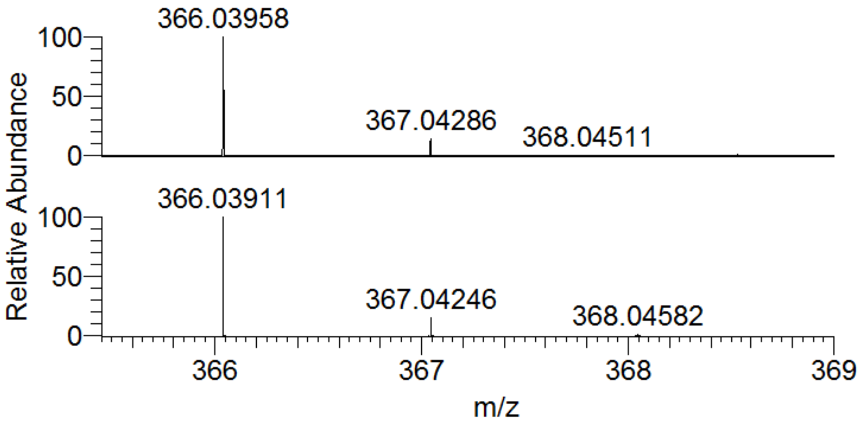

Figure S6. Experimental (top) and calculated (bottom) isotopic pattern of the species $[V^{IV}O(dhp)_2+Na]^+$ (m/z = 366.04).

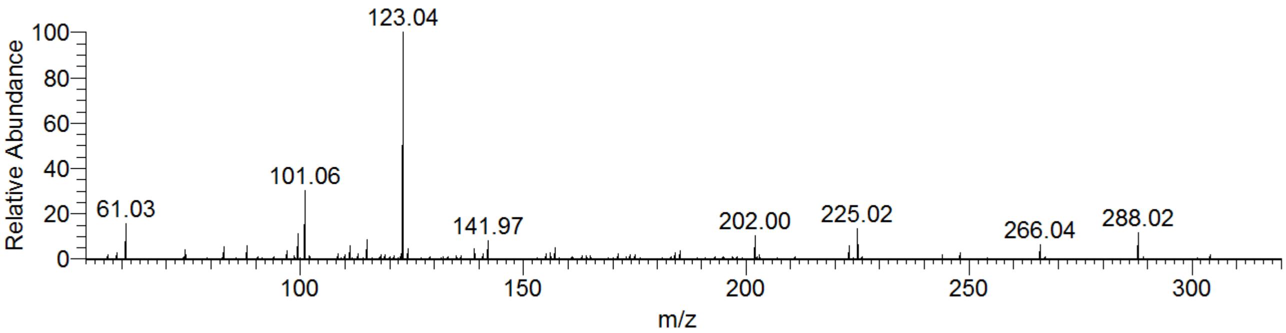

Figure S7. ESI-MS spectrum recorded in positive mode at pH 5.50 on the system containing $[V^{IV}O(acac)_2]$ in ultrapure LC-MS water with a V concentration of 5.0 μM.

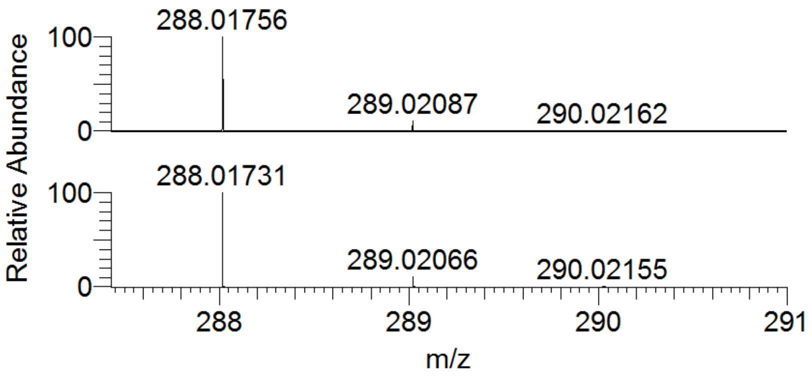

Figure S8. Experimental (top) and calculated (bottom) isotopic pattern of the species $[V^{IV}O(acac)_2+Na]^+$ (m/z = 288.02).

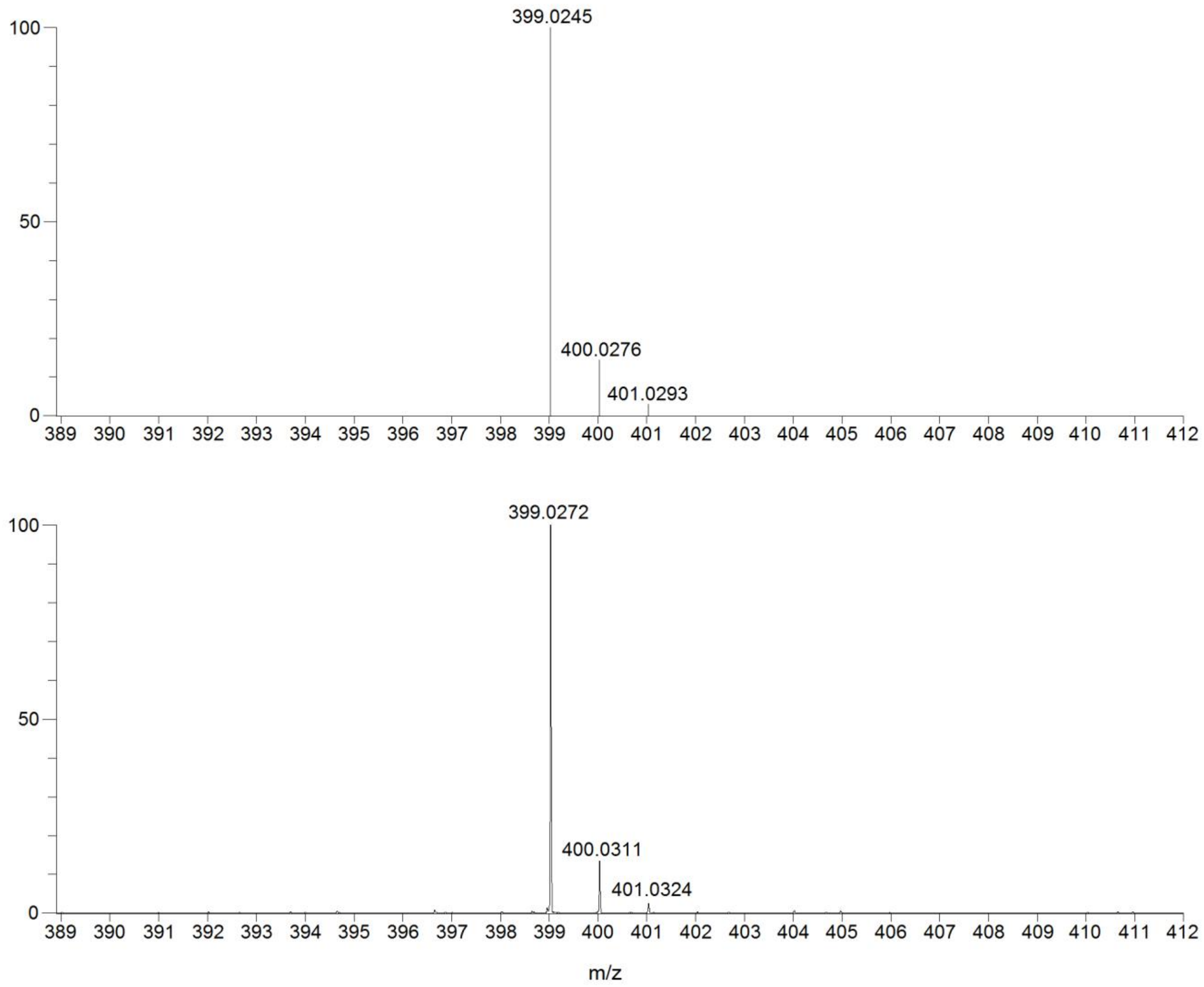

Figure S9. Experimental (top) and calculated (bottom) isotopic pattern of the species $[V^{V}(hidpa)_2]^{-}$ (m/z = 399.02).

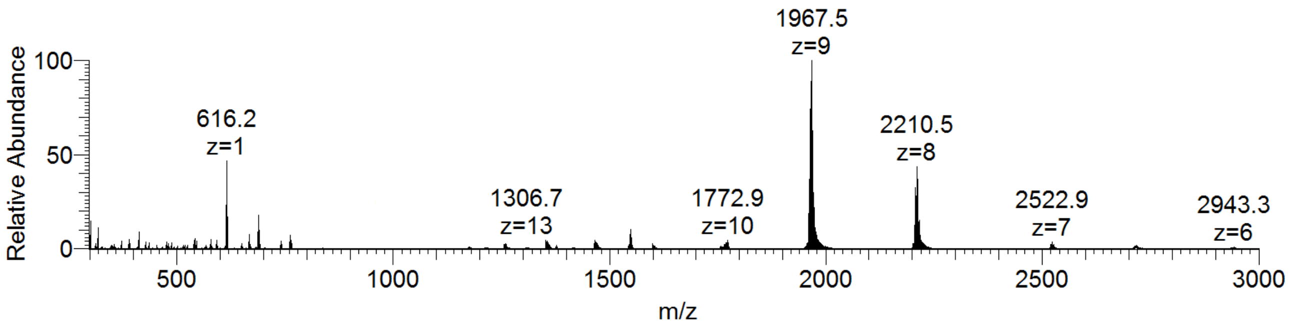

Figure S10. ESI-MS spectrum of myoglobin (concentration 5 μM).

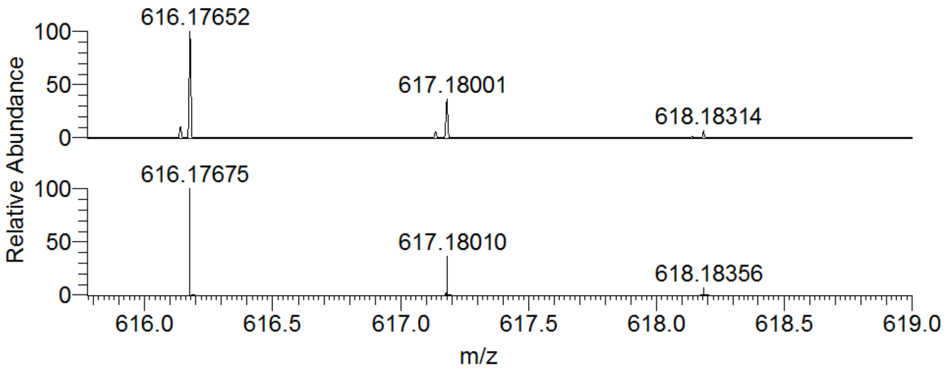

Figure S11. Experimental (top) and calculated (bottom) isotopic pattern for the peak of $[Fe^{III}heme]^{+}$ group ($C_{34}H_{32}FeN_4O_4$) revealed at m/z = 616.18 in the ESI-MS spectrum of myoglobin (concentration 5 µM).

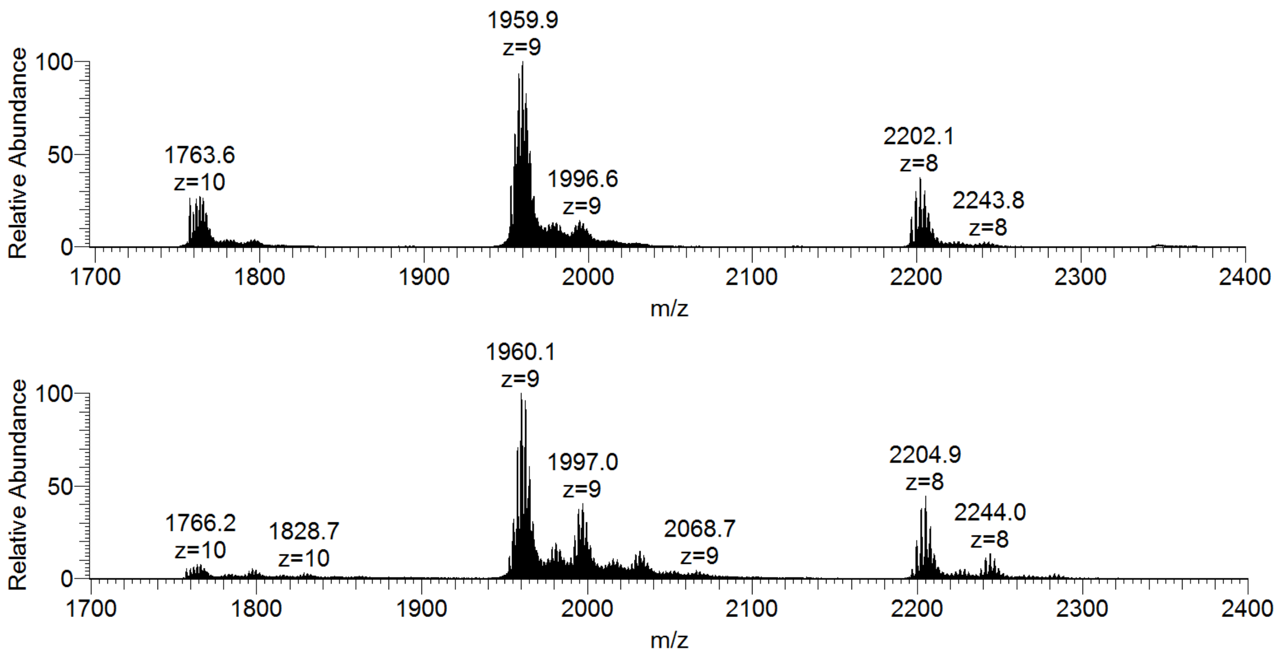

Figure S12. ESI-MS spectra recorded on the system containing [$V^{IV}O(pic)_2(H_2O)$] and myoglobin (5 µM): molar ratio 3/1 (top) and 5/1 (bottom).

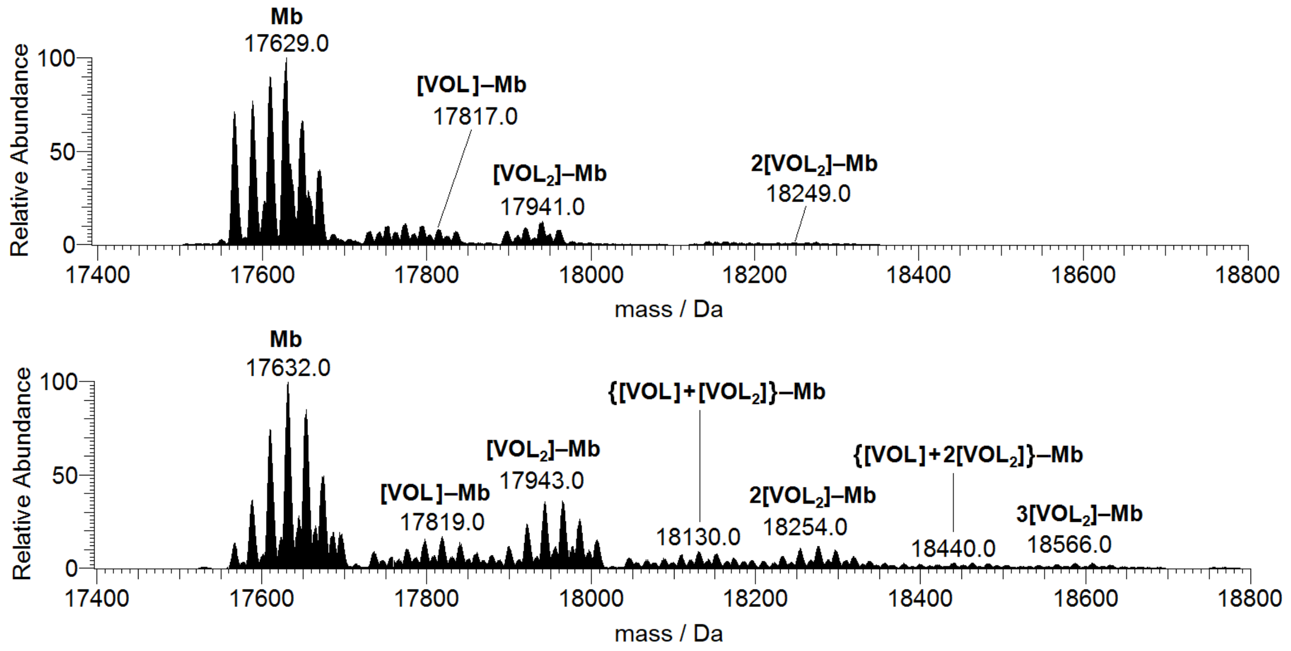

Figure S13. Deconvoluted ESI-MS spectra recorded on the system containing $[V^{IV}O(pic)_2(H_2O)]$ and myoglobin (5 µM): molar ratio 3/1 (top) and 5/1 (bottom). L indicates the picolinato ligand.

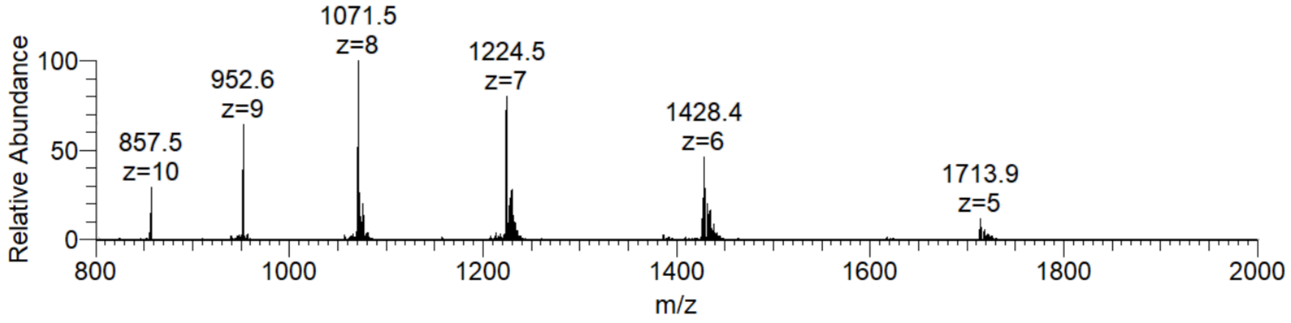

Figure S14. ESI-MS spectrum of ubiquitin (concentration 5 µM).

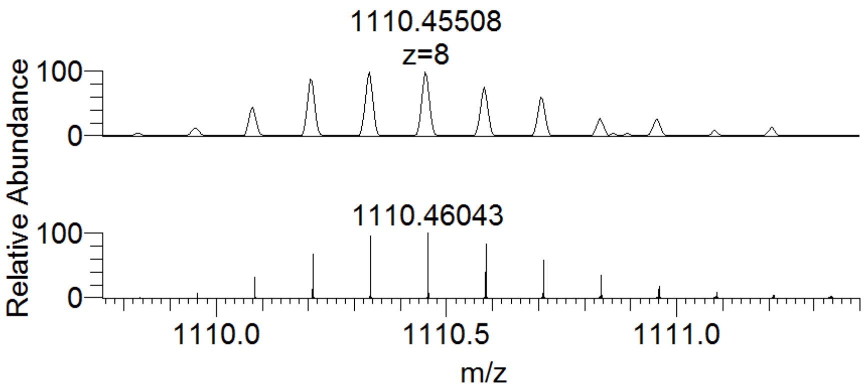

Figure S15. Experimental (top) and calculated (bottom) isotopic pattern for the peak of $[V^{IV}O(pic)_2]$–Ub (m/z = 1110.46, z = 8) with formula $C_{390}H_{638}N_{107}O_{123}SV$ detected in the ESI-MS spectrum of the system containing $[V^{IV}O(pic)_2(H_2O)]$ and ubiquitin with ratio 3/1 and Ub concentration of 5 μM.

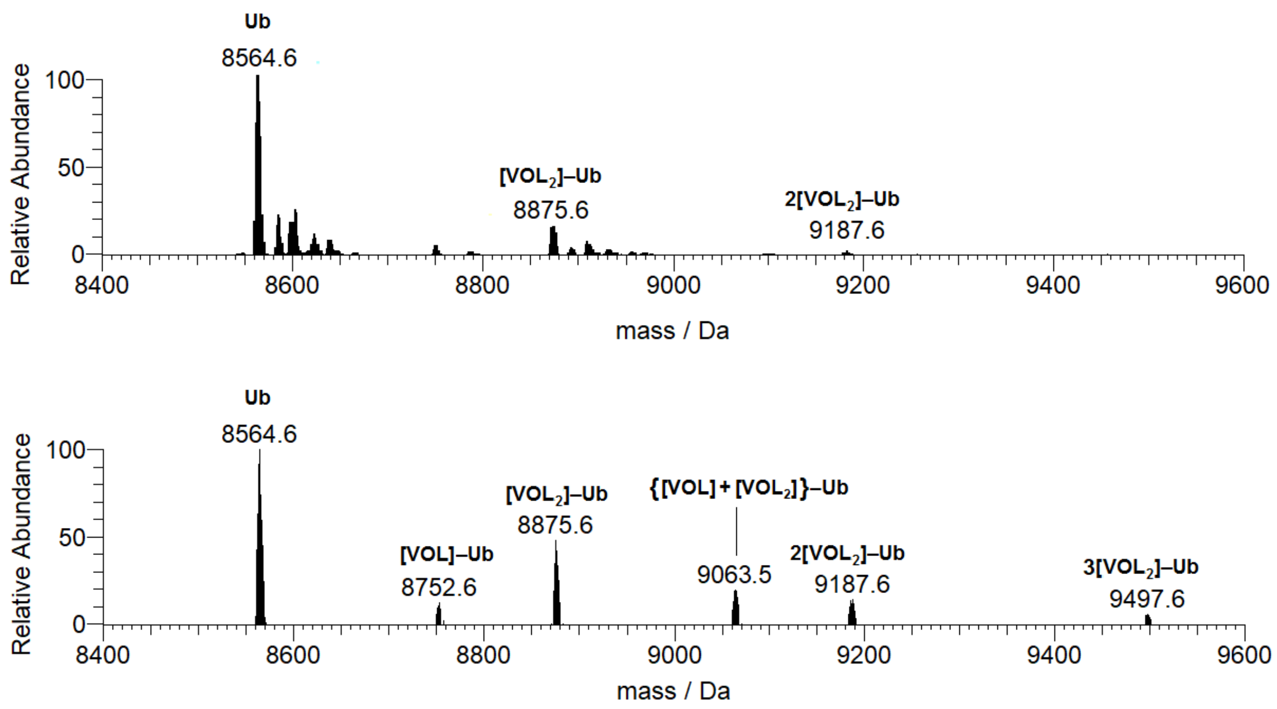

Figure S16. Deconvoluted ESI-MS spectra recorded on the system containing $[V^{IV}O(pic)_2(H_2O)]$ and ubiquitin (50 µM): molar ratio 3/1 (top) and 5/1 (bottom). L indicates the picolinato ligand.

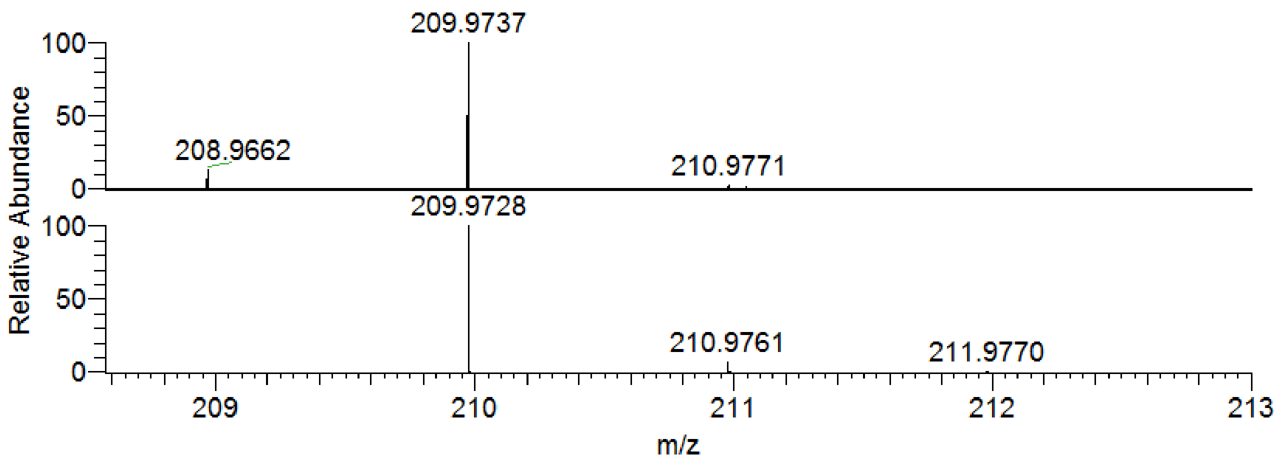

Figure S17. Experimental (top) and calculated (bottom) isotopic pattern of $[V^{IV}O(ma)(H_2O)]^+$ detected in the ESI-MS spectrum of the system containing $[V^{IV}O(ma)_2]$ (150 µM) and lysozyme (50 µM) (m/z = 209.97).

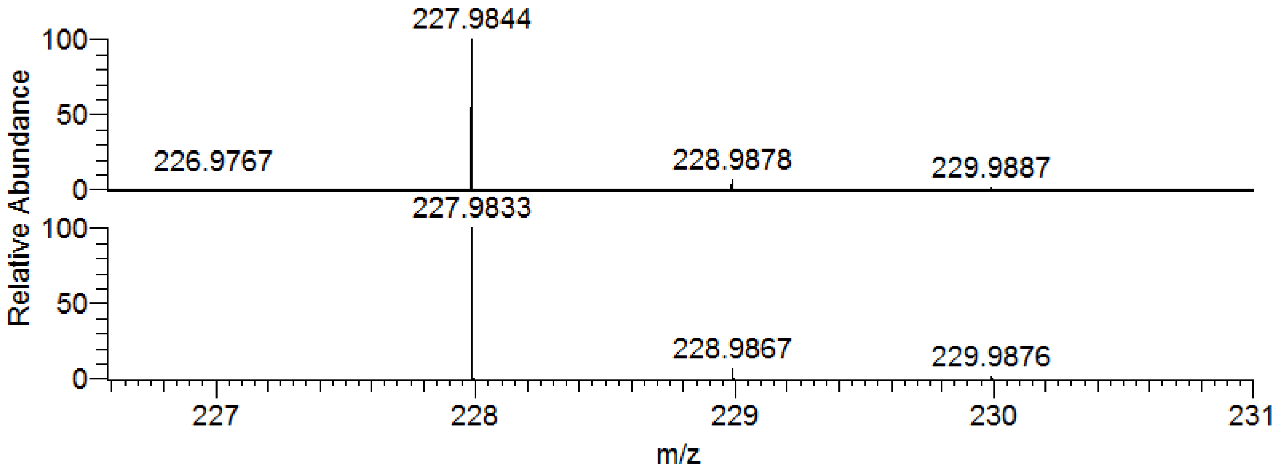

Figure S18. Experimental (top) and calculated (bottom) isotopic pattern of $[V^{IV}O(ma)(H_2O)_2]^+$ detected in the ESI-MS spectrum of the system containing $[V^{IV}O(ma)_2]$ (150 μM) and lysozyme (50 μM) (m/z = 227.98).

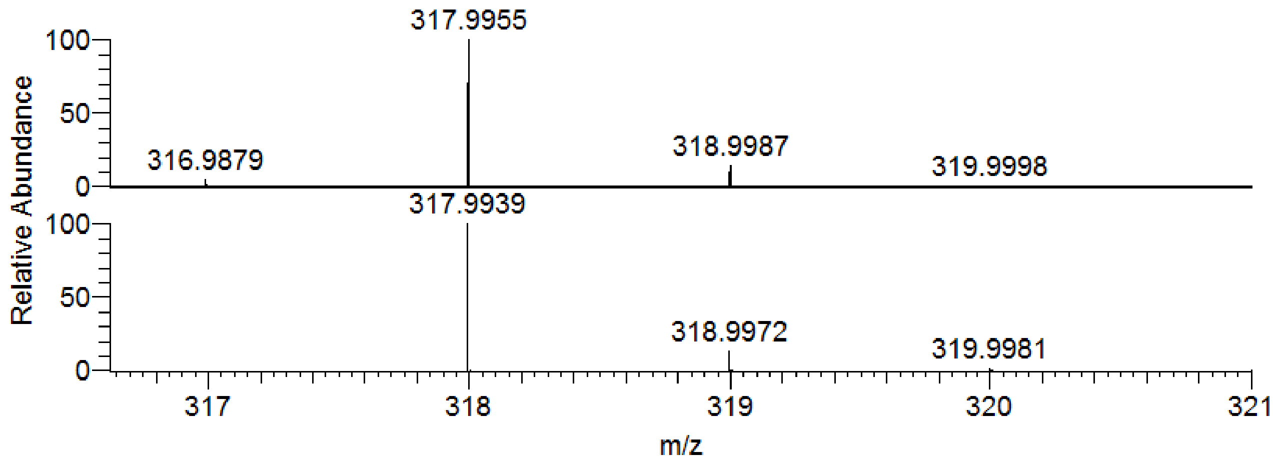

Figure S19. Experimental (top) and calculated (bottom) isotopic pattern of $[V^{IV}O(ma)_2+H]^+$, detected in the ESI-MS spectrum of the system containing $[V^{IV}O(ma)_2]$ (150 μM) and lysozyme (50 μM) (m/z = 318.00).

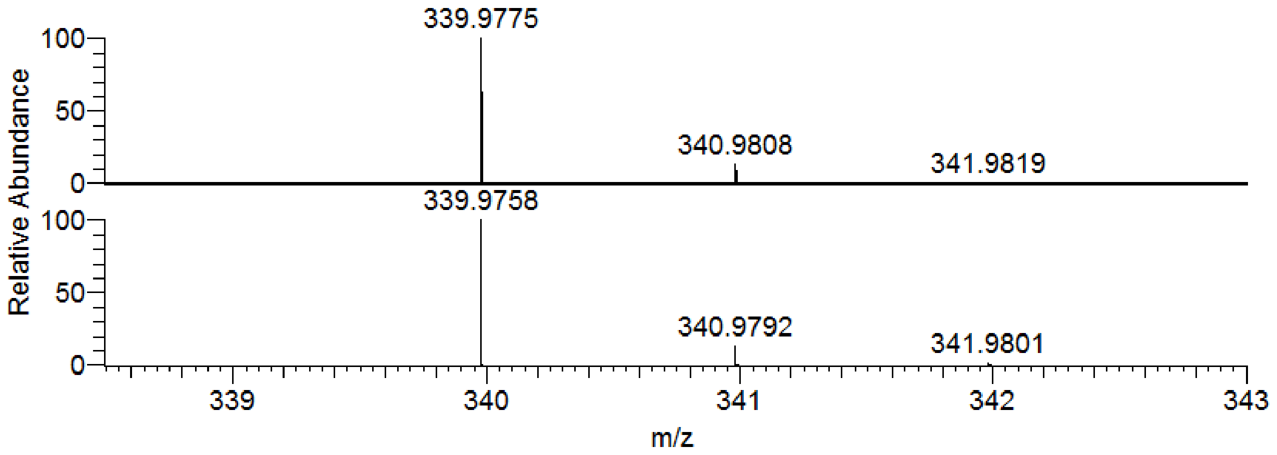

Figure S20. Experimental (top) and calculated (bottom) isotopic pattern of $[V^{IV}O(ma)_2+Na]^+$ detected in the ESI-MS spectrum of the system containing $[V^{IV}O(ma)_2]$ (150 μM) and lysozyme (50 μM) (m/z = 339.98).

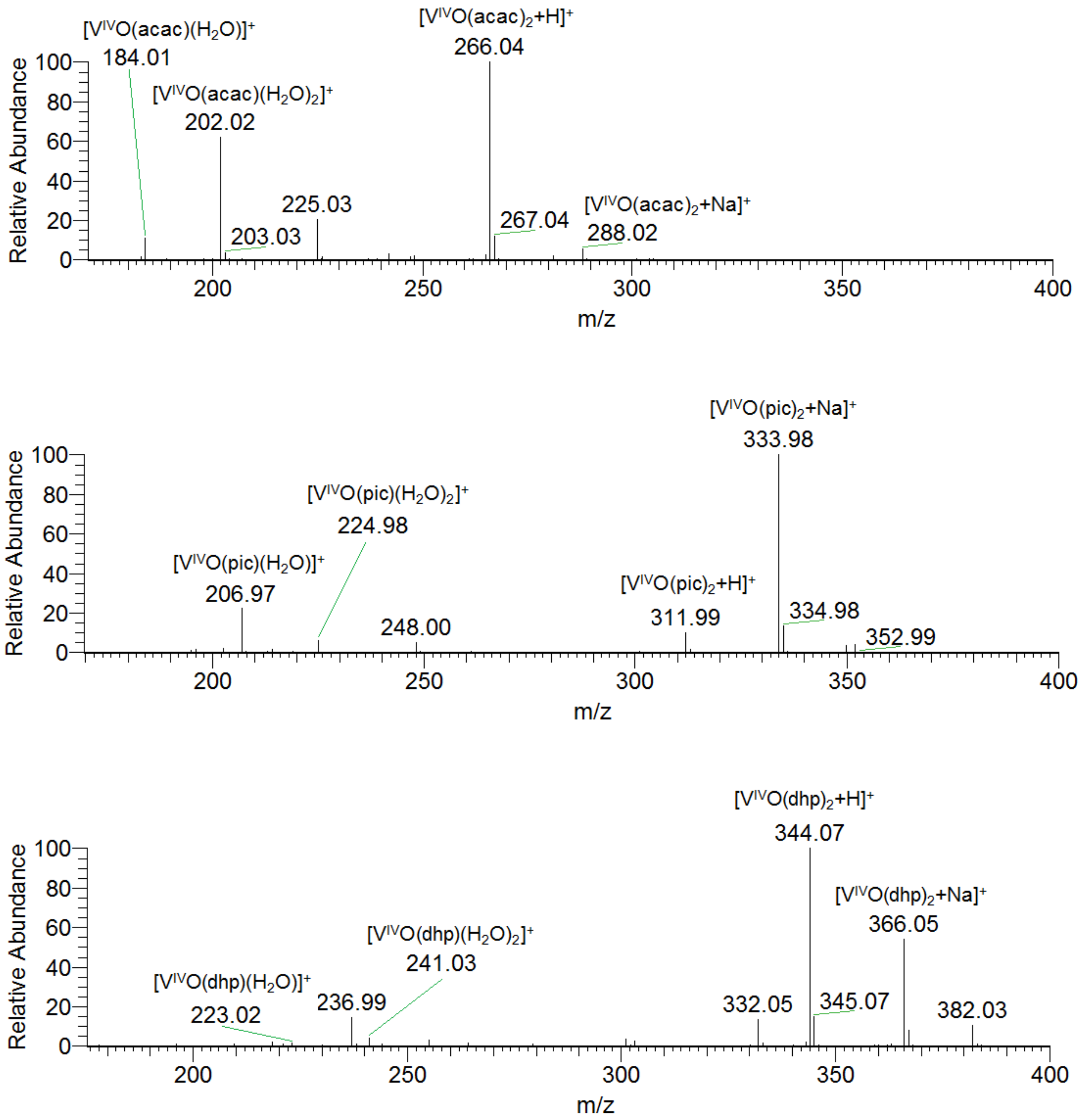

Figure S21. Region in the m/z range 180-400 of the ESI-MS spectra recorded on the systems containing [$V^{IV}O(acac)_2$] (150 μM) and lysozyme (50 μM) (top), [$V^{IV}O(pic)_2(H_2O)_2$] (150 μM) and lysozyme (50 μM) (centre), and [$V^{IV}O(dhp)_2$] (150 μM) and lysozyme (50 μM) (bottom).

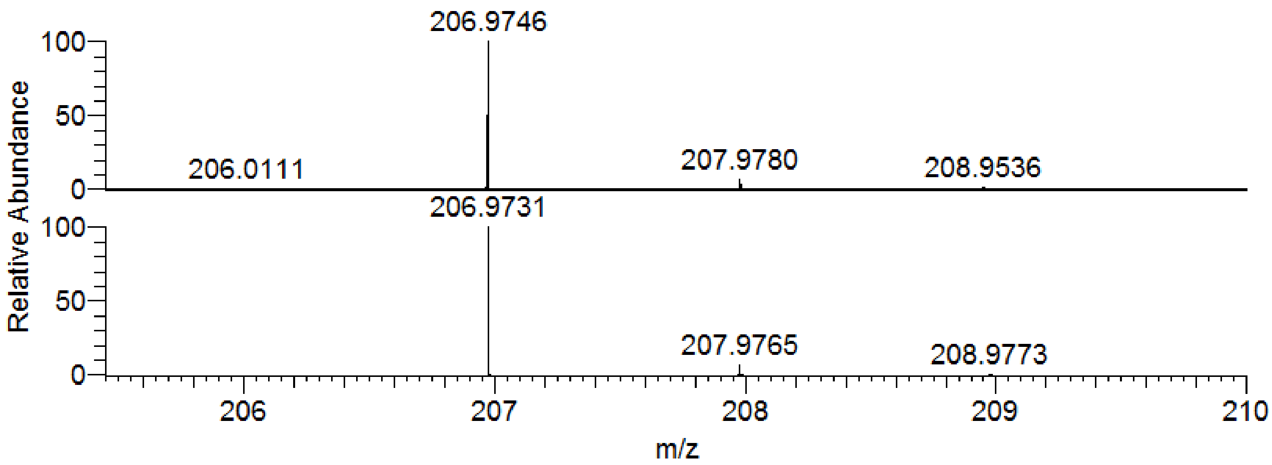

Figure S22. Experimental (top) and calculated (bottom) isotopic pattern of $[V^{IV}O(pic)(H_2O)]^+$ detected in the ESI-MS spectrum of the system containing $[V^{IV}O(pic)_2(H_2O)]$ (150 μM) and lysozyme (50 μM) (m/z = 206.97).

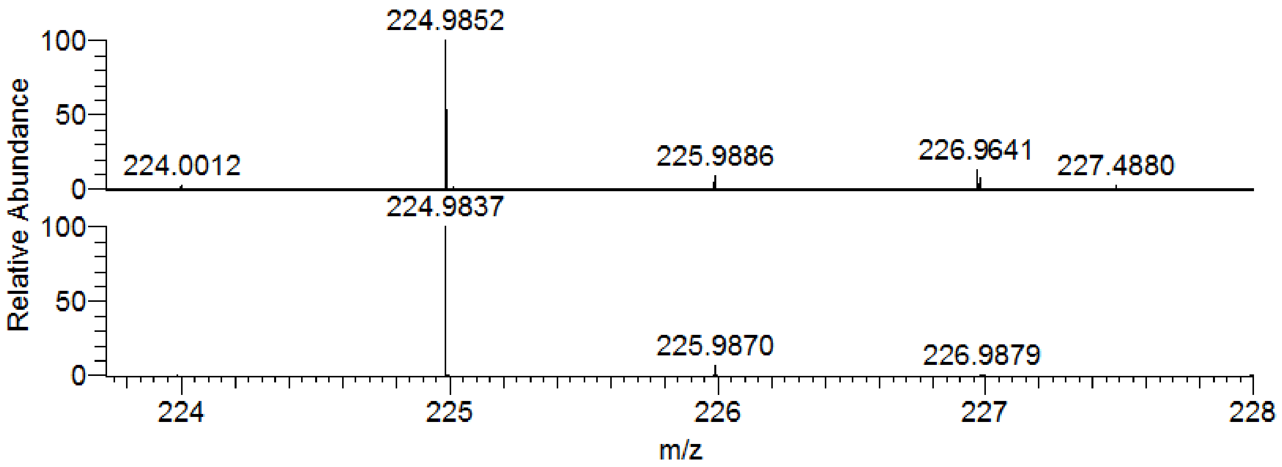

Figure S23. Experimental (top) and calculated (bottom) isotopic pattern of $[V^{IV}O(pic)(H_2O)_2]^+$ detected in the ESI-MS spectrum of the system containing $[V^{IV}O(pic)_2(H_2O)]$ (150 µM) and lysozyme (50 µM) (m/z = 224.99).

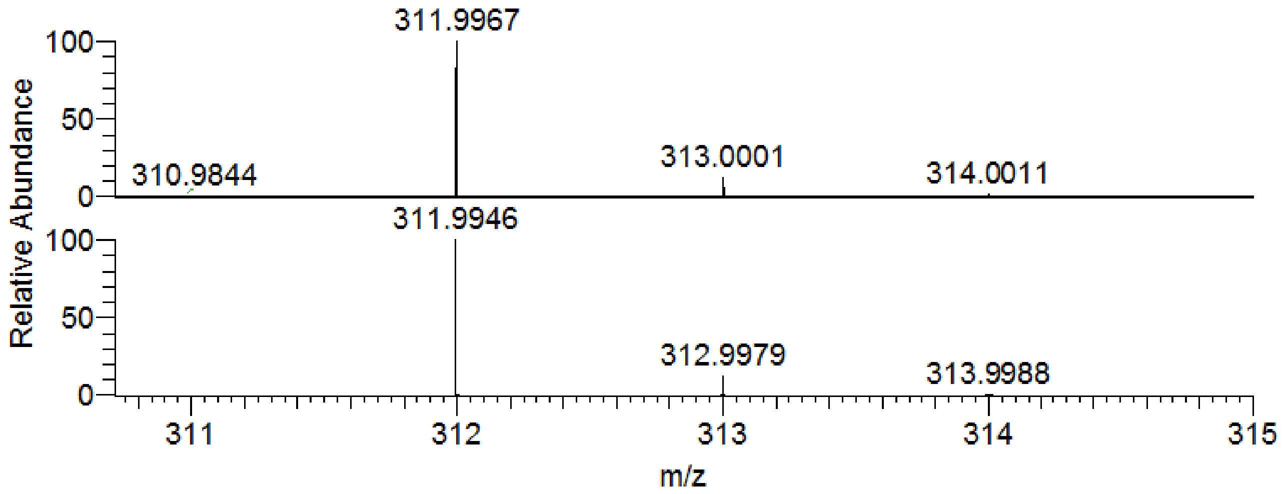

Figure S24. Experimental (top) and calculated (bottom) isotopic pattern of $[V^{IV}O(pic)_2+H]^+$, detected in the ESI-MS spectrum of the system containing $[V^{IV}O(pic)_2(H_2O)]$ (150 μM) and lysozyme (50 μM) (m/z = 312.00).

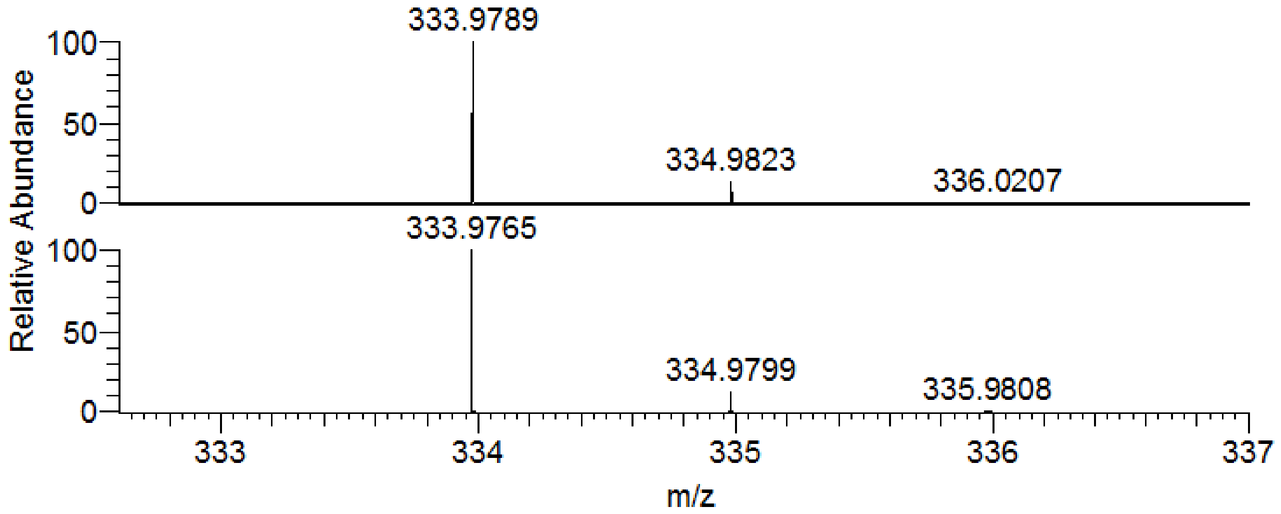

Figure S25. Experimental (top) and calculated (bottom) isotopic pattern of $[V^{IV}O(pic)_2+Na]^+$, detected in the ESI-MS spectrum of the system containing $[V^{IV}O(pic)_2(H_2O)]$ (150 μM) and lysozyme (50 μM) (m/z = 333.98).

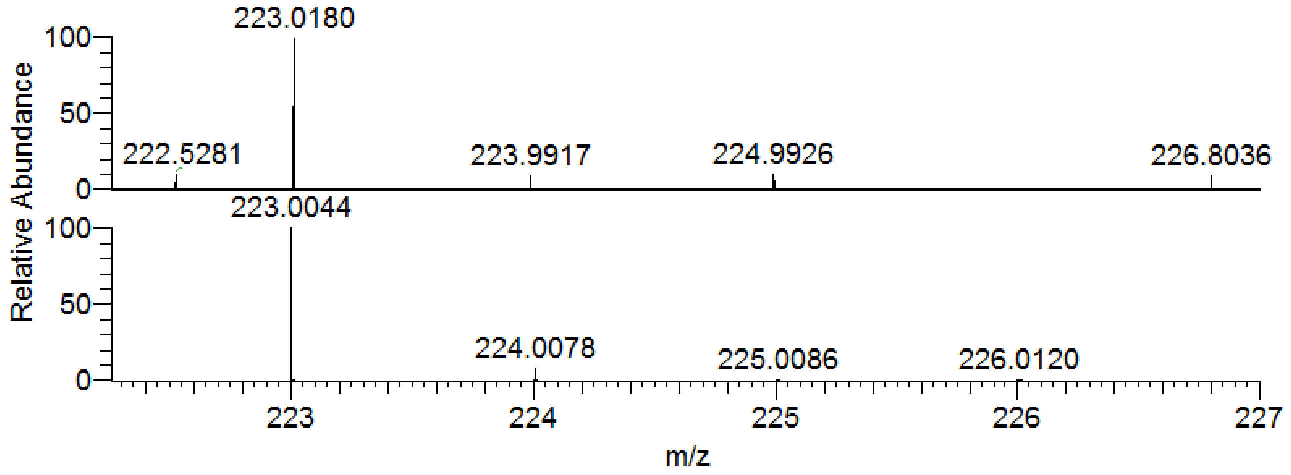

Figure S26. Experimental (top) and calculated (bottom) isotopic pattern of $[V^{IV}O(dhp)(H_2O)]^+$ detected in the ESI-MS spectrum of the system containing $[V^{IV}O(dhp)_2]$ (150 μM) and lysozyme (50 μM) (m/z = 223.02).

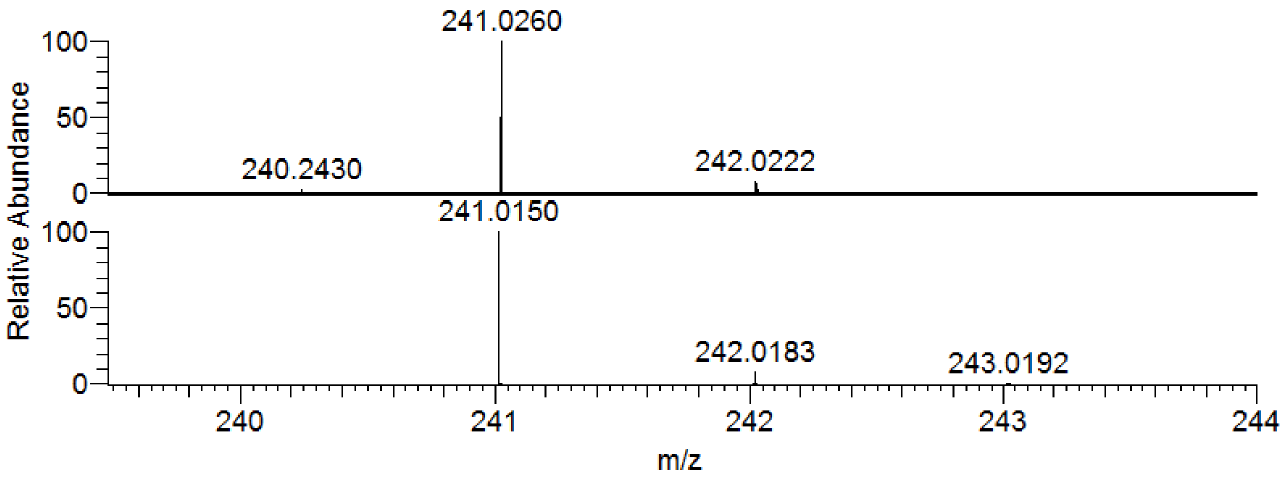

Figure S27. Experimental (top) and calculated (bottom) isotopic pattern of $[V^{IV}O(dhp)(H_2O)_2]^+$ detected in the ESI-MS spectrum of the system containing $[V^{IV}O(dhp)_2]$ (150 μM) and lysozyme (50 μM) (m/z = 241.03).

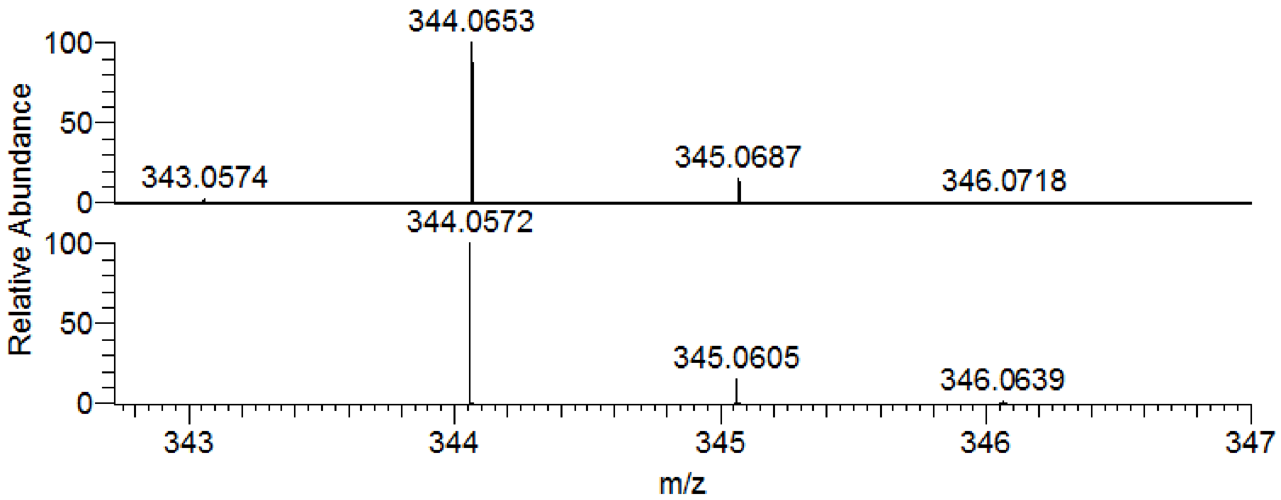

Figure S28. Experimental (top) and calculated (bottom) isotopic pattern of $[V^{IV}O(dhp)_2+H]^+$, detected in the ESI-MS spectrum of the system containing $[V^{IV}O(dhp)_2]$ (150 μM) and lysozyme (50 μM) (m/z = 344.07).

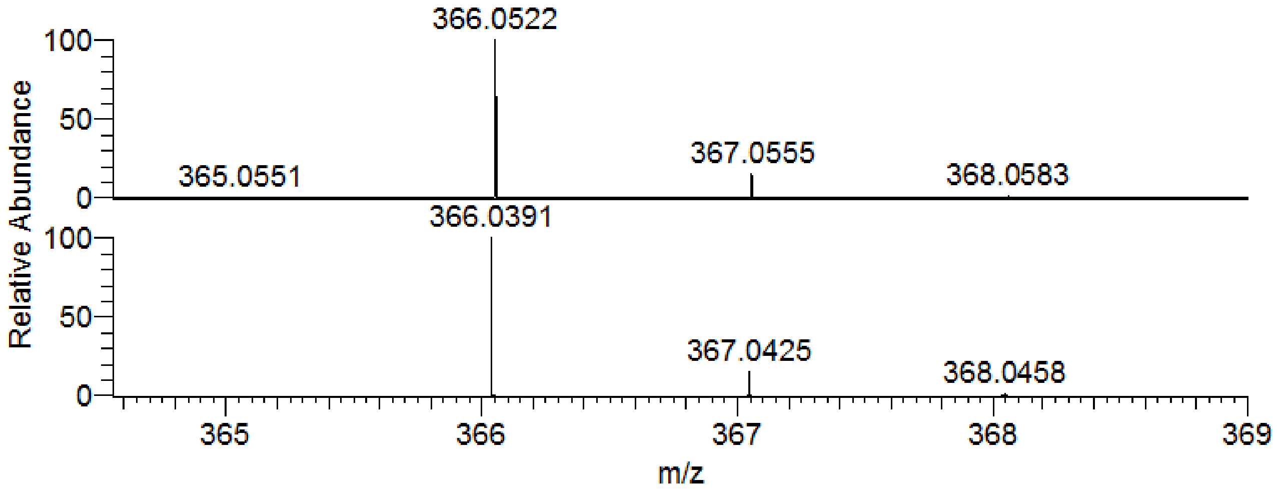

Figure S29. Experimental (top) and calculated (bottom) isotopic pattern of $[V^{IV}O(dhp)_2{+}Na]^+$, detected in the ESI-MS spectrum of the system containing $[V^{IV}O(dhp)_2]$ (150 µM) and lysozyme (50 µM) (m/z = 366.05).

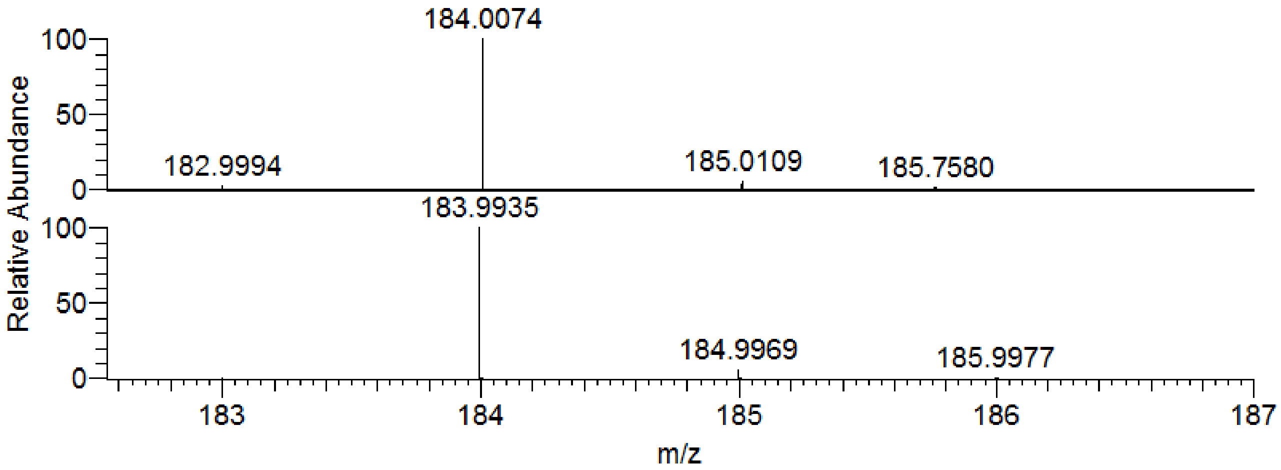

Figure S30. Experimental (top) and calculated (bottom) isotopic pattern of $[V^{IV}O(acac)(H_2O)]^+$ detected in the ESI-MS spectrum of the system containing $[V^{IV}O(acac)_2]$ (150 μM) and lysozyme (50 μM) (m/z = 184.01).

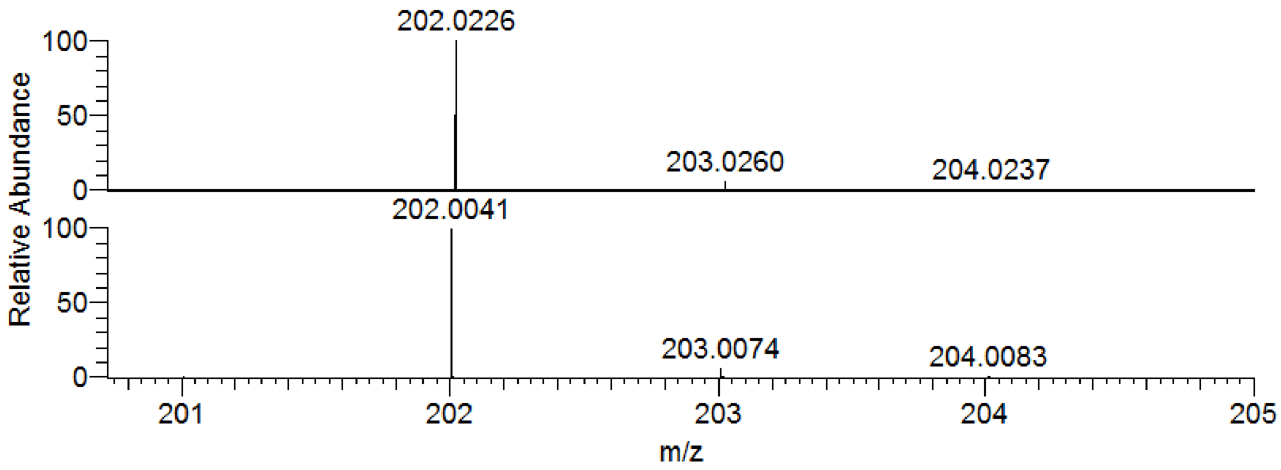

Figure S31. Experimental (top) and calculated (bottom) isotopic pattern of $[V^{IV}O(acac)(H_2O)_2]^+$ detected in the ESI-MS spectrum of the system containing $[V^{IV}O(acac)_2]$ (150 µM) and lysozyme (50 µM) (m/z = 202.02).

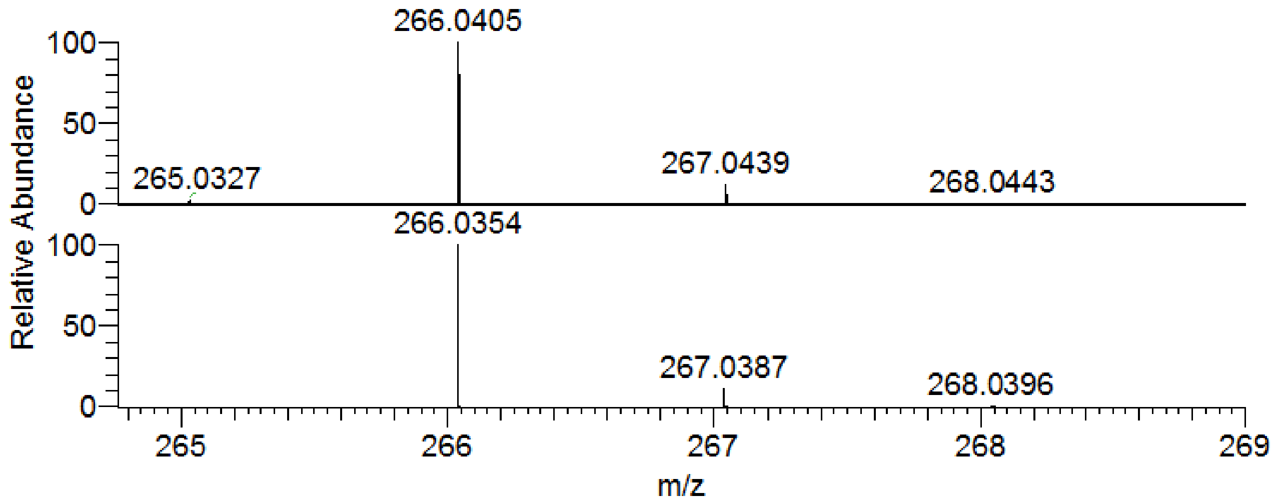

Figure S32. Experimental (top) and calculated (bottom) isotopic pattern of $[V^{IV}O(acac)_2+H]^+$ detected in the ESI-MS spectrum of the system containing $[V^{IV}O(acac)_2]$ (150 µM) and lysozyme (50 µM) (m/z = 266.04).

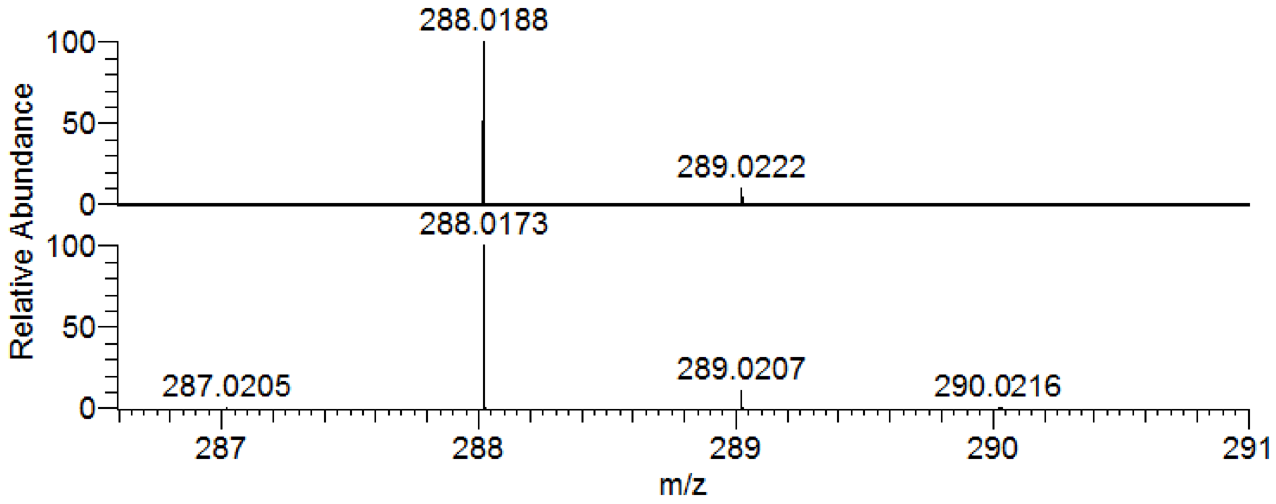

Figure S33. Experimental (top) and calculated (bottom) isotopic pattern of $[V^{IV}O(acac)_2+Na]^+$ detected in the ESI-MS spectrum of the system containing $[V^{IV}O(acac)_2]$ (150 μM) and lysozyme (50 μM) (m/z = 288.02).

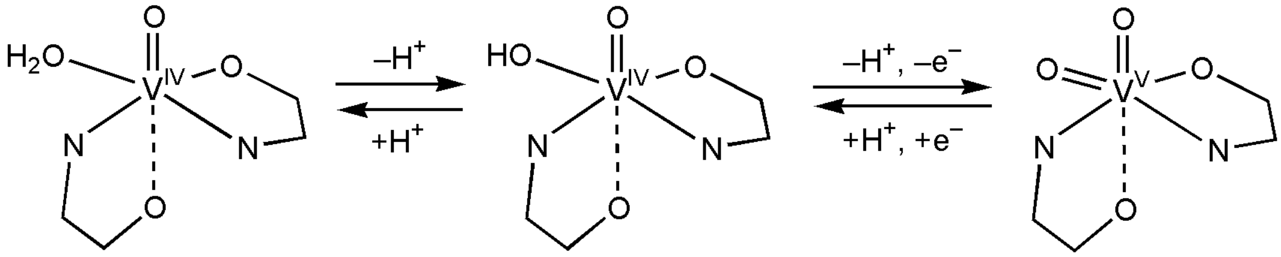

Scheme S1. Oxidation of *cis*-$V^{IV}O(H_2O)^{2+}$ to *cis*-$V^{V}O_2^{+}$ moiety.
